# A novel CDC25A/DYRK2 regulatory switch modulates cell cycle and survival

**DOI:** 10.1038/s41418-021-00845-5

**Published:** 2021-08-06

**Authors:** Maribel Lara-Chica, Alejandro Correa-Sáez, Rafael Jiménez-Izquierdo, Martín Garrido-Rodríguez, Francisco J. Ponce, Rita Moreno, Kimberley Morrison, Chiara Di Vona, Krisztina Arató, Carla Jiménez-Jiménez, Rosario Morrugares, M. Lienhard Schmitz, Susana de la Luna, Laureano de la Vega, Marco A. Calzado

**Affiliations:** 1 Instituto Maimónides de Investigación Biomédica de Córdoba (IMIBIC), Córdoba, Spain; 2 Departamento de Biología Celular, Fisiología e Inmunología, Universidad de Córdoba, Córdoba, Spain; 3 Hospital Universitario Reina Sofía, Córdoba, Spain; 4 Division of Cellular Medicine, School of Medicine, University of Dundee, Scotland, UK; 5 Centre for Genomic Regulation (CRG), The Barcelona Institute of Science and Technology (BIST), Barcelona, Spain; 6 Centro de Investigación Biomédica en Red en Enfermedades Raras (CIBERER), Barcelona, Spain; 7 Institute of Biochemistry, Justus-Liebig-University, Member of the German Center for Lung Research, Giessen, Germany; 8 Universitat Pompeu Fabra (UPF), Barcelona, Spain; 9 Institució Catalana de Recerca i Estudis Avançats (ICREA), Barcelona, Spain

**Keywords:** Proteins, Cell biology, Proteomics

## Abstract

The cell division cycle 25A (CDC25A) phosphatase is a key regulator of cell cycle progression that acts on the phosphorylation status of Cyclin–Cyclin-dependent kinase complexes, with an emergent role in the DNA damage response and cell survival control. The regulation of CDC25A activity and its protein level is essential to control the cell cycle and maintain genomic integrity. Here we describe a novel ubiquitin/proteasome-mediated pathway negatively regulating CDC25A stability, dependent on its phosphorylation by the serine/threonine kinase DYRK2. DYRK2 phosphorylates CDC25A on at least 7 residues, resulting in its degradation independent of the known CDC25A E3 ubiquitin ligases. CDC25A in turn is able to control the phosphorylation of DYRK2 at several residues outside from its activation loop, thus affecting DYRK2 localization and activity. An inverse correlation between DYRK2 and CDC25A protein amounts was observed during cell cycle progression and in response to DNA damage, with CDC25A accumulation responding to the manipulation of DYRK2 levels or activity in either physiological scenario. Functional data show that the pro-survival activity of CDC25A and the pro-apoptotic activity of DYRK2 could be partly explained by the mutual regulation between both proteins. Moreover, DYRK2 modulation of CDC25A expression and/or activity contributes to the DYRK2 role in cell cycle regulation. Altogether, we provide evidence suggesting that DYRK2 and CDC25A mutually control their activity and stability by a feedback regulatory loop, with a relevant effect on the genotoxic stress pathway, apoptosis, and cell cycle regulation.

## Introduction

The cell division cycle-25 (CDC25) family of dual-specificity phosphatases has a key role in controlling cell cycle progression by acting on the phosphorylation status of cyclin-dependent kinases (CDKs) [1, 2]. CDC25 family members in humans (CDC25A, CDC25B, and CDC25C) are also part of cellular checkpoints of DNA damage [3], being CDC25A the most studied. CDC25A dephosphorylates several CDKs regulating not only early G1/S transition but also late G2/M [4]. CDC25A is considered an oncogene, as its overexpression correlates with tumoral development in many cancer types [5–9] and is associated with alterations in relevant oncogenic pathways [10, 11].

CDC25A dysregulation is often related to cell cycle alterations and apoptosis inhibition, supporting the importance of tight control. CDC25A protein levels are subject to post-translational regulation, particularly during the cell cycle by the ubiquitin–proteasome-dependent pathway. While Cyclin E/CDK2-mediated phosphorylation increases CDC25A phosphatase activity during the S phase, Cyclin B/CDK1 mediated phosphorylation at Ser17 and Ser115 prevents CDC25A degradation after entering mitosis [12]. In addition, other kinases such as Checkpoint kinase (CHK) 1 and CHK2 target CDC25A for induced protein degradation during the cell cycle and also in response to DNA damage [13–15]. Although the identification of the controlling CDC25A phosphorylation events is of great interest, this area has not yet been well explored.

Dual-specificity tyrosine-phosphorylation-regulated kinase 2 (DYRK2) is a Ser/Thr kinase belonging to the CMGC group, whose activity depends on its autophosphorylation on a tyrosine residue within the activation loop (T-loop) [16, 17]. DYRK2 is a key regulator of DNA damage response pathways and stress signals, and it has been implicated in several human cancers with both oncogenic and antitumor suppressor activities (reviewed in [18–20]). To date, more than twenty DYRK2 substrates have been identified, including c-Jun, c-Myc, Nuclear factor of activated T-cells (NFAT) family members, NOTCH1, heat shock factor 1 (HSF1), and p53 [21–25]. Furthermore, DYRK2-associated molecular functions have been linked to tumor cells such as G1/S transition [21], epithelial-mesenchymal-transition [26], and stemness of cancer cells [27].

Here, we describe DYRK2 as a new CDC25A regulator. DYRK2 phosphorylates CDC25A and facilitates its ubiquitination-mediated degradation, controlling CDC25A levels in response to DNA damage. In turn, DYRK2 is a CDC25A substrate, thus regulating DYRK2 phosphorylated status and its kinase activity. In summary, we describe a novel regulatory mechanism that has important implications for the control of the cell cycle and survival.

## Results

### CDC25A protein levels are modulated by DYRK2

**Fig. 1:**
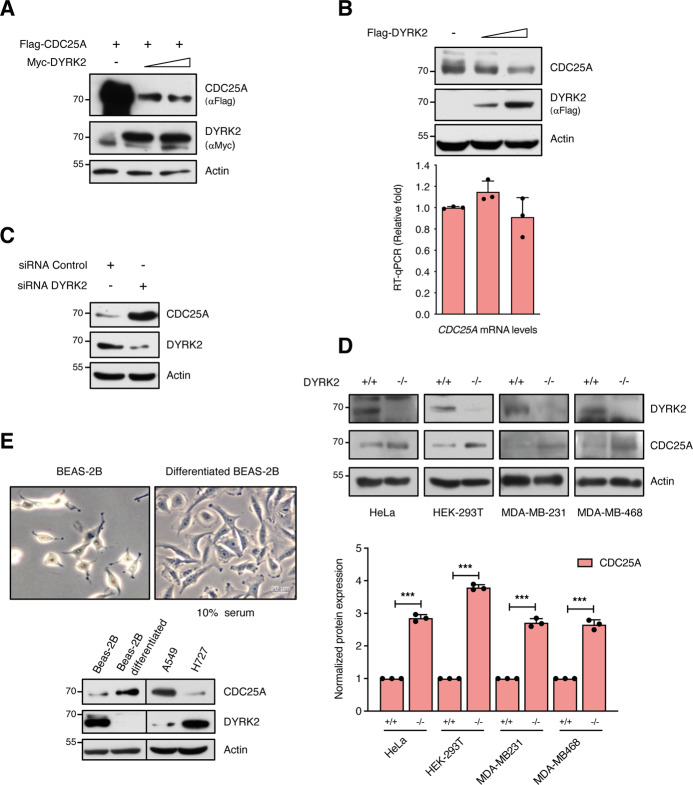
DYRK2 expression affects the stability of CDC25A. **A** Extracts from HEK-293T expressing Flag-CDC25A and Myc-DYRK2 by transient transfection were analyzed by WB. A representative experiment is shown (*n* = 3). **B** HeLa cells were transfected to express increasing amounts of DYRK2 and endogenous CDC25A protein and mRNA and levels were analyzed by immunoblot (upper panel, a representative experiment of three performed) and RT-qPCR (lower panel), respectively. The bar graph shows relative mRNA levels with mock cells set as 1 (mean ± SD, *n* = 3). **C** HEK-293T cells were transfected with DYRK2 or control siRNAs, and DYRK2 and CDC25A were analyzed after 4 days in culture (representative blot of two independent experiments performed). **D** CDC25A and DYRK2 levels in the indicated cell lines WT (+/+) and knock-out (−/−) for DYRK2. A representative blot of three independent experiments is shown. The bar graph shows the quantification of CDC25A (normalized by Actin) with that of the cell lines WT (+/+) set as 1 (mean ± SD, *n* = 3; ***P < 0.001). **E** Top panel: illustrative images of the BEAS-2B differentiation model, in which human bronchial epithelial cells (BEAS-2B; left panel) undergo squamous differentiation in response to serum (right panel). Lower panel: Total cell extracts from the indicated cell lines were analyzed by WB with specific DYRK2 and CDC25A antibodies. The vertical line indicates that the images are from different blots. A representative blot of three independent experiments is shown.

To identify new DYRK2 substrates, we performed a kinase array in which 1024 peptides were incubated with DYRK2 (data not shown), emerging CDC25A within the top 30 hits. Given its essential role in cell-cycle progression, we focused on studying the functional interaction between the two proteins. Co-expression studies showed that CDC25A protein levels were reduced in the presence of DYRK2 (Fig. 1A), with similar results on endogenous CDC25A (Fig. 1B). The DYRK2-dependent effect was post-transcriptional since *CDC25A* mRNA amounts were not affected. The effect is specific to CDC25A because the accumulation of the other CDC25 paralogs (B and C) was not altered (Supplementary Fig. 1A). Other members of the DYRK subfamily were also evaluated, but only DYRK1B behaved as DYRK2 (Supplementary Fig. 1B). To validate the results, we undertook a loss-of-function approach. DYRK2 depletion by siRNA substantially increased CDC25A protein levels (Fig. 1C) and led to an extension of its half-life (Supplementary Fig. 1C). The results were further evaluated in different DYRK2 knock-out cell lines generated by CRISPR/Cas9. Elimination of DYRK2 led to the increase in CDC25A levels in all the cell lines analyzed (Fig. 1D). In summary, these results show that DYRK2 acts on CDC25A at the protein level by reducing its stability.

Higher expression of CDC25A protein has been observed in lung adenocarcinoma cells than in normal lung fibroblasts [28]. Furthermore, we have previously shown that DYRK2 levels respond to the differentiation of BEAS-2B cells, human bronchial epithelial cells that undergo squamous differentiation in response to serum [29] (Fig. 1E). Indeed, an inverse correlation between both proteins was observed in these cells and in other lung cancer cell lines (Fig. 1E). Notably, the more differentiated the BEAS-2B cells were and a lower DYRK2 expression was detected, a higher expression of CDC25A was observed (Fig. 1E).

### DYRK2 kinase activity is required for CDC25A degradation by the ubiquitin/proteasome system

**Fig. 2:**
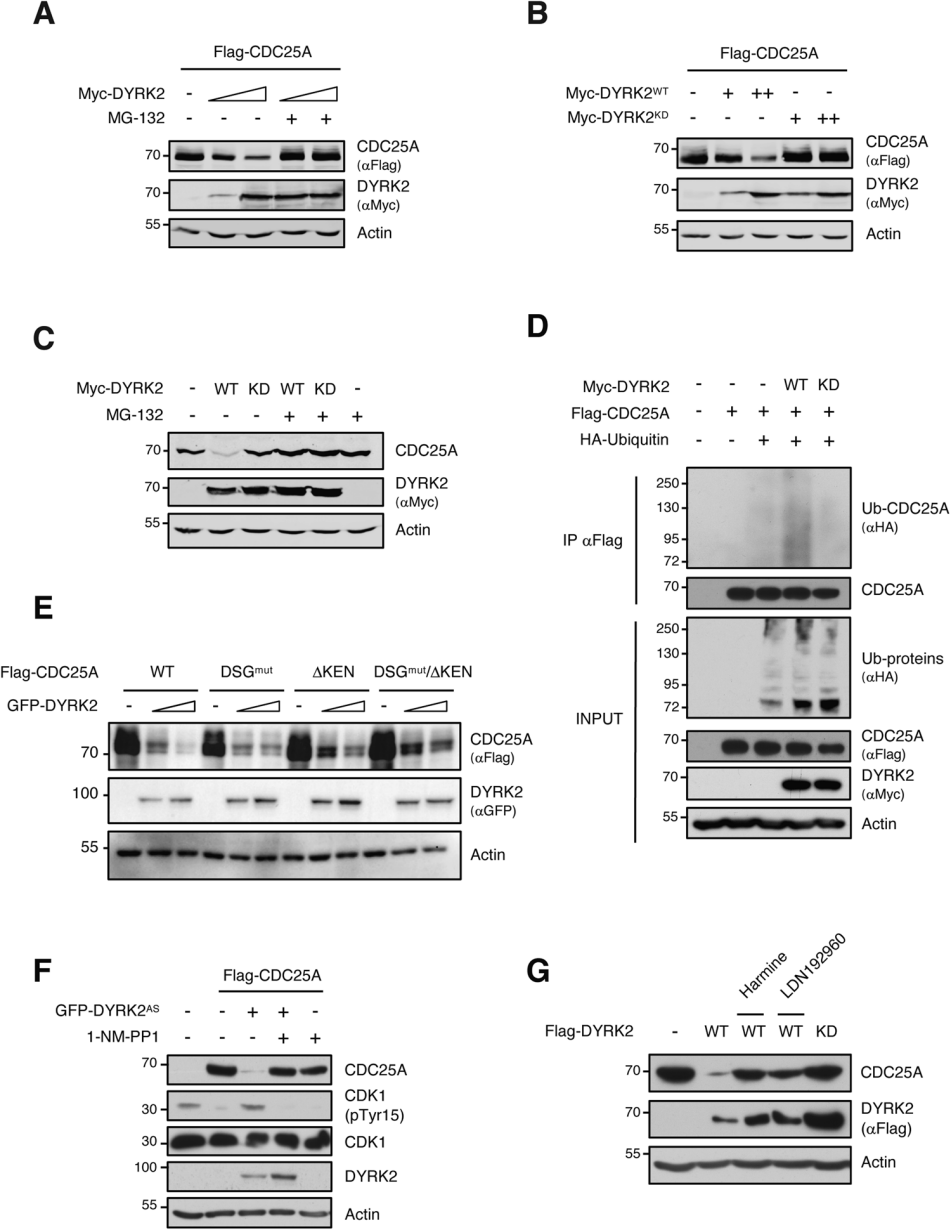
CDC25A degradation by DYRK2 is proteasome and DYRK2 kinase activity-dependent. **A** Transfected HEK-293T cells with the indicated plasmids were treated with the proteasome inhibitor MG-132 (10 μM) for 12 h and proteins analyzed by WB. **B**, **C** Extracts from HEK-293T cells (**B**) or differentiated BEAS-2B cells (**C**) transfected with the indicated plasmids were analyzed by WB. In panel (**C**), the cells were treated with MG-132 (10 μM) or vehicle for 12 h. **D** Transfected HeLa cells with the indicated plasmids were treated with MG-132 (10 μM) for 12 h and lysed under denaturing conditions. Flag-tagged CDC25A was purified by anti-Flag immunoprecipitation and ubiquitinated CDC25A detected by WB. **E** Cell extracts from HEK-293T expressing CDC25A-WT, a DSG mutant (S88A), a mutant with the KEN-box deleted (ΔKEN), or a mutant with both mutations together or not with GFP-DYRK2 were analyzed by WB. A representative experiment of three performed is shown. **F** Transfected HeLa cells were treated with 1-NM-PP1 (3 μM) for 3 h, before analysis of protein extracts by WB. In all panels, a representative blot of two or three independent experiments performed is shown. **G** Transfected HEK-293T cells with the indicated plasmids were treated with Harmine (7 μM) or LDN192960 (10 μM) for 12 h and 2 h, respectively, before analysis of protein extracts by WB. In all panels, a representative blot of two or three independent experiments performed is shown.

As CDC25A phosphorylation is often accompanied by proteasome-dependent degradation [4], we analyzed whether DYRK2 was modulating CDC25A by this mechanism. CDC25A was co-expressed with DYRK2 in the presence of the proteasome inhibitor MG-132, which rescued the CDC25A levels (Fig. 2A and Supplementary Fig. 2A, B). This protective effect was detected not only on ectopically expressed CDC25A but also on the endogenous protein (Fig. 2C). Next, we analyzed the impact of DYRK2 kinase activity, and whereas DYRK2-WT overexpression led to a reduction in CDC25A, no effect was observed with a DYRK2 kinase-dead (KD) mutant (Fig. 2A). This effect was also confirmed in differentiated BEAS-2B cells (Supplementary Fig. 2A), H727 cells (Supplementary Fig. 2B), and by a reduction in CDC25A half-life in cells expressing DYRK2-WT as compared to cells expressing DYRK2-KD (Supplementary Fig. 2C). Next, we examined whether DYRK2 triggered changes in CDC25A ubiquitination. Overexpression of DYRK2 led to an increase in CDC25A polyubiquitination, which was not observed in the presence of DYRK2-KD (Fig. 2D), further supporting a proteasome-dependent pathway.

The candidate ubiquitin ligase complexes leading to the degradation of CDC25A include SCF^βTRCP^ and APC/C[4, 30], which need to recognize specific residues phosphorylated within the DSG motif or bind the KEN motif, respectively. To evaluate the importance of these, we generated CDC25A DSG and KEN mutants. The two CDC25A mutant proteins responded similarly as the WT protein to either DYRK2 overexpression (Fig. 2E) or depletion (Supplementary Fig. 2D), indicating that the DYRK2-mediated degradation pathway proceeds independently of these two E3 ligases. Altogether, these results demonstrate that DYRK2 negatively regulates CDC25A levels through a new ubiquitin, proteasome, and DYRK2 kinase activity-dependent process.

Finally, to test the relevance of DYRK2 using an independent experimental approach, we used an analog DYRK2 mutant (DYRK2-AS) sensitive to pyrazolo[3,4-*d*]pyrimidine-based inhibitors [25]. DYRK2-AS-mediated decrease of CDC25A levels did not occur in the presence of the AS-kinase 1NM-PP1 inhibitor, with a strong impact on CDC25A-dependent activities as shown by the CDK1 phosphorylation levels at (Fig. 2F). We also evaluated the effect of DYRK2 small molecule inhibitors. Treatment with the DYRK inhibitor Harmine [31] or with the potent selective DYRK2 inhibitor LDN192960 [32] abrogated CDC25A degradation (Fig. 2G). Altogether, these experiments demonstrate that DYRK2 modulates CDC25A protein accumulation in a kinase-dependent manner.

### DYRK2 co-localizes with CDC25A

**Fig. 3:**
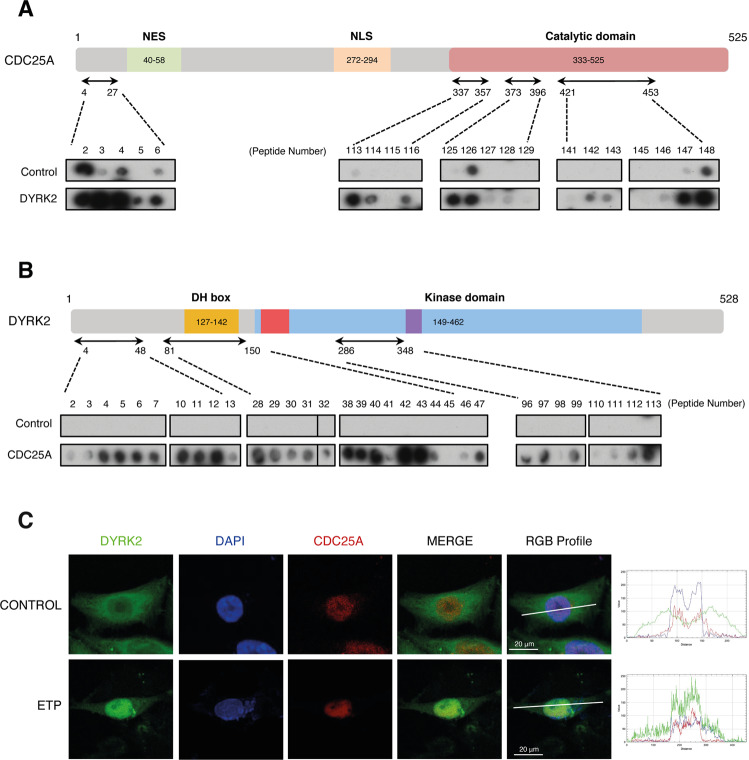
DYRK2 co-localizes and interacts with CDC25A. **A**, **B** Results of the mapping experiments in CDC25A (**A**) and DYRK2 (**B**) using a peptide library for each protein. The schemes show the functional domains and the regions responsible for interaction between the two proteins (NES nuclear export signal, NLS nuclear localization signal, DH box DYRK-homology box). The dot-blot results of the analysis for the screen (see “Methods”) are also shown only for the peptides giving the positive signal and with unfused GST used as control. **C** CHO cells were treated with etoposide (ETP; 10 μM) or vehicle for 24 h, and with MG-132 (10 μM) for the last 4 h to promote CDC25A stabilization. Nuclear DNA was stained with DAPI. RGB profiles correspond to the fluorescence intensity through the white line indicating DYRK2 and CDC25A endogenous localization in both control and DNA damage conditions. A representative picture with overlapping localization in yellow is shown. (Pearson’s coefficient below threshold = 0.07 and Manders’ coefficients of *A* = 0.781; *B* = 0.669).

Given the functional relationship between DYRK2 and CDC25A, it is reasonable to consider that they are interaction partners. We performed peptide array experiments to identify their binding regions. The results showed that CDC25A might have four potential interacting sites for DYRK2, one in the N-terminus and three contiguous regions within the catalytic domain (Fig. 3A and Supplementary Fig. 3A). The experiments also identified three binding regions within DYRK2: one in the non-catalytic N-terminus, a second one overlapping with the DYRK Homology (DH)-box, and the NAPA domain, and several contact points nearby the activation segment (Fig. 3B and Supplementary Fig. 3A). In addition, we cannot exclude the existence of further interacting surfaces in the context of the properly folded proteins. Despite these results, we have not succeeded in detecting the interaction by co-immunoprecipitation assays, suggesting that it is transient and/or involves a small pool of any of the two proteins. As an alternative, we analyzed DYRK2 and CDC25A subcellular localization using confocal microscopy, which showed partial co-localization of the two proteins in the nucleus, increasing considerably after Etoposide stimulation (Fig. 3C; Supplementary Fig. 3B, C).

### DYRK2 phosphorylates CDC25A

**Fig. 4:**
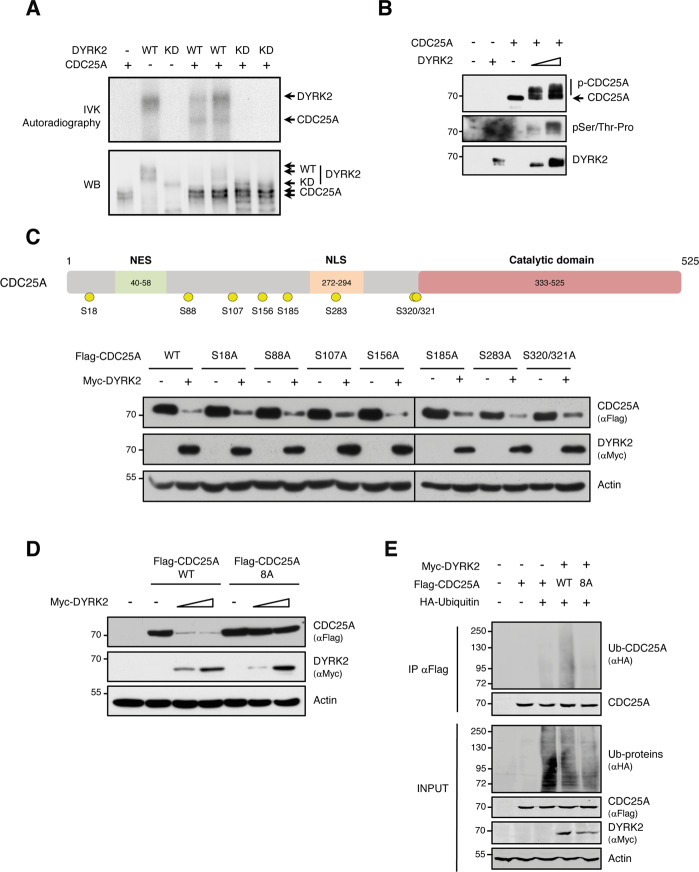
CDC25A is phosphorylated by DYRK2. **A** Purified CDC25A protein was incubated with GST-DYRK2-WT, GST-DYRK2-KD, or GST alone. Phosphorylation was determined by autoradiography, while CDC25A and DYRK2 were detected by WB with antibodies to GST and CDC25A, respectively. **B** IVK assay performed with purified DYRK2 and CDC25A proteins. CDC25A phosphorylation was determined by WB with a phospho-S/T-Pro antibody, while total protein levels of DYRK2 and CDC25A were assessed by WB. **C** TOP: Schematic CDC25A representation showing different functional regions and the phosphorylated amino acids detected (in yellow). BOTTOM: Extracts from transfected HEK-293T cells with plasmids to express Flag-CDC25A-WT or the indicated mutant versions either alone or in the presence of Myc-DYRK2 were examined by WB. We show a representative experiment of three performed. The vertical lines indicate that the images are from different blots. **D** Extracts from cells expressing Flag-CDC25A-WT or the mutant version with eight Ser-to-Ala changes (Flag-CDC25A-8A), either alone or together with Myc-DYRK2 were analyzed by WB. We show a representative experiment of three performed. **E** Transfected HeLa cells with the indicated plasmids were treated with MG-132 (10 μM) for 12 h and lysed under denaturing conditions. Flag-tagged CDC25A-WT and the mutant version CDC25A-8A were purified by anti-Flag immunoprecipitation and ubiquitinated CDC25A detected by WB.

The dependence on DYRK2 kinase activity suggests the possibility of CDC25A being its substrate. Thus, we carried out radioactive IVK (in vitro kinase) assays with purified proteins. Incorporation of ^32^P was detected both in DYRK2, as a result of autophosphorylation, and in CDC25A indicating that this protein is a direct substrate of DYRK2 (Fig. 4A). In agreement, incubation of CDC25A with purified DYRK2 resulted in the appearance of CDC25A species with lower electrophoretic mobility, which were detected with a phospho-Ser/Thr-Pro antibody (Fig. 4B).

To identify the CDC25A sites phosphorylated by DYRK2, we used mass spectrometry analysis on purified CDC25A phosphorylated in vitro. The analysis showed that CDC25A was phosphorylated in at least 7 residues: S18, S107, S156, S185, S283, S320, and S321 (Supplementary Fig. 4A). To establish a functional relationship, we generated non-phosphorylatable mutants (serine to alanine) for each of them, including S88, which had previously been described as relevant for the control of CDC25A accumulation [13]. The expression of all single-site mutants was reduced by DYRK2 (Fig. 4C), suggesting that none of these phosphorylation sites controls CDC25A stability alone. In contrast, a CDC25A variant with the eight residues mutated to alanine (CDC25A-8A) was completely resistant to the DYRK2-induced degradation (Fig. 4D). In agreement and in contrast to CDC25A WT, the mutant protein did not polyubiquitinate in the presence of DYRK2 (Fig. 4E). All these results indicate that DYRK2 directly phosphorylates CDC25A at several residues and that DYRK2 phosphorylation at more than one of these sites is required to induce CDC25A degradation.

### DYRK2 phosphorylation status outside the T-loop impacts DYRK2 activity

**Fig. 5:**
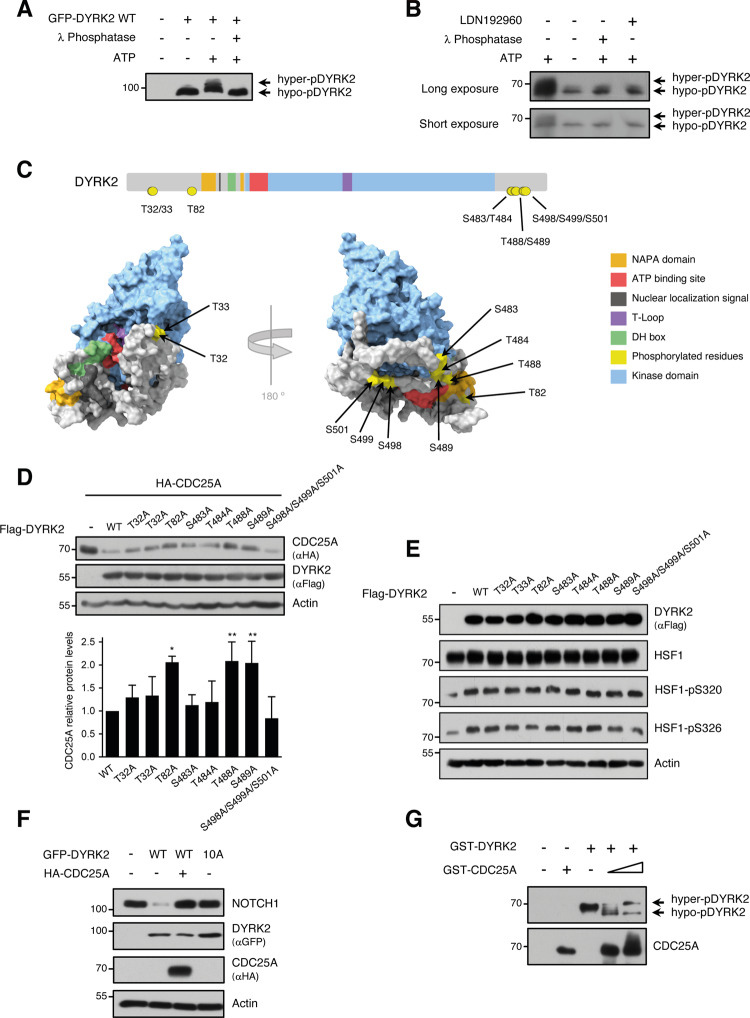
Autophosphorylation of DYRK2. **A** GFP-DYRK2-WT expressed in HEK-293T cells was purified by anti-GFP immunoprecipitation and used in IVK assays. DYRK2 was detected by WB. We show a representative experiment of two performed. **B** IVK assays were performed with purified DYRK2 in the absence or presence of λ-phosphatase and LDN192960 (10 μM). DYRK2 was detected by WB. **C** TOP: Schematic DYRK2 representation showing different functional regions (see the color code) and the phosphorylated amino acids (in yellow). BOTTOM: Space-filling models of DYRK2 shown from different angles, using the color code to identify the different domains and the phosphorylated amino acids (in yellow). **D** The stability of HA-CDC25A was analyzed in HeLa cells co-expressing Flag-DYRK2-WT or the indicated mutants. The bar graph shows the quantification of HA-CDC25A (normalized by the Actin signal) relative to the protein levels in the presence of DYRK2-WT set as 1 (mean ± SD, *n* = 3; *P < 0.05, **P < 0.01). **E** Extracts from HeLa cells expressing Flag-DYRK2-WT or the indicated mutant versions were analyzed by WB to detect endogenous HSF1 and two of the DYRK2-dependent HSF1 phosphosites. We show a representative experiment of two performed. **F** Extracts from HeLa cells expressing HA-CDC25A together with GFP-DYRK2-WT or with a mutant version containing all putative DYRK2 autophosphorylation residues mutated to alanine (10 A) were analyzed by WB. A representative experiment is shown out of three independent experiments. **G** In vitro phosphatase assay performed with purified CDC25A on autophosphorylated purified DYRK2. Both proteins were detected by WB.

The changes in electrophoretic mobility observed for DYRK2 in the IVK assays (Fig. 4A) could indicate that it is autophosphorylated at several residues in addition to the activation loop [17, 33]. Autophosphorylation outside the T-loop has been detected for DYRK1A, DYRK1B, and DYRK3 [34–36], but no information on DYRK2 is available yet. To test this hypothesis, we performed IVK assays in the presence of λ phosphatase or the DYRK2 inhibitor LDN192960. Both treatments abrogated the changes in electrophoretic mobility induced by the phosphorylation reaction (Fig. 5A, B). DYRK2 from IVK reactions was analyzed by MS, which led to the identification of several phosphosites besides the residue Y309 within the T-loop: T32, T33, T82, S483, T484, T488, S489, and S498/499/501 (Fig. 5C and Supplementary Fig. 5A). The autophosphorylation of a DYRK2 mutant with all these residues mutated to alanine showed no changes in electrophoretic mobility (Supplementary Fig. 5B), suggesting that the analysis identified most of the DYRK2 autophosphosites responsible for the mobility shift. The phosphosites are located at the non-catalytic N- and C-terminal regions and none of them are within regions already known to play functional roles. However, when these residues were placed in the DYRK2 3D structure, all of them, except T82, are present on the surface and in proximity to the active site (Fig. 5C). The results show that DYRK2 is autophosphorylated on at least 10 residues besides the Tyr of the activation loop.

To evaluate the impact of these phosphorylation events on DYRK2 properties, we generated DYRK2 versions with each phosphorylatable residue mutated to alanine. Mutations in T484, T488, and S498/499/501 resulted in impaired expression levels of DYRK2 in transient transfection experiments (Supplementary Fig. 5C). The mutations also resulted in changes in the subcellular distribution for some of the mutants. Thus, DYRK2-T32A and -T484A were mostly nuclear and DYRK2-T82A and -S489A were found distributed in both the nucleus and the cytoplasm in contrast with the preferred cytoplasmic localization of DYRK2-WT  (Supplementary Fig. 5D). Finally, we assessed the impact of DYRK2 phosphorylation on the catalytic activity of each mutant by different assays using similar protein expression for all of them: on peptide-based IVKs to evaluate the intrinsic enzymatic activity and on CDC25A stability as well as on other known DYRK2 substrates such as HSF-1 [25] and NOTCH1 [24]. Results with the peptide-IVKs showed a slight reduction in catalytic activity for the DYRK2-S498/499/501A mutant, and an increase in activity for the DYRK2-T32/33A mutant (Supplementary Fig. 5E), and more importantly none of them impaired DYRK2 intrinsic kinase activity. Regarding the experiments on specific substrates, the mutations T82A, T488A, and S489A decreased the DYRK2 ability to regulate CDC25A accumulation compared with the WT version (Fig. 5D). In contrast, no significant differences were observed on HSF-1 phosphorylation (Fig. 5E). Finally, a DYRK2 version with all the residues changed to alanine was unable to induce changes in NOTCH1 stability (Fig. 5F), supporting the importance of the autophosphorylation sites in regulating DYRK2 activity.

Interestingly, CDC25A caused an increase in DYRK2 electrophoretic mobility (Fig. 5G), suggesting that DYRK2 might be a substrate of the CDC25A phosphatase. More importantly, CDC25A restrained the ability of DYRK2 to control NOTCH1 protein levels in the same way as the non-phosphorylatable DYRK2 mutant does (Fig. 5F), indicating that the regulation of the DYRK2 phosphorylation status by CDC25A results in functional outputs. These results led us to suggest that DYRK2 and CDC25A mutually control their activity and stability by a feedback regulatory loop.

### DYRK2 regulates effects mediated by CDC25A protein stabilization

**Fig. 6:**
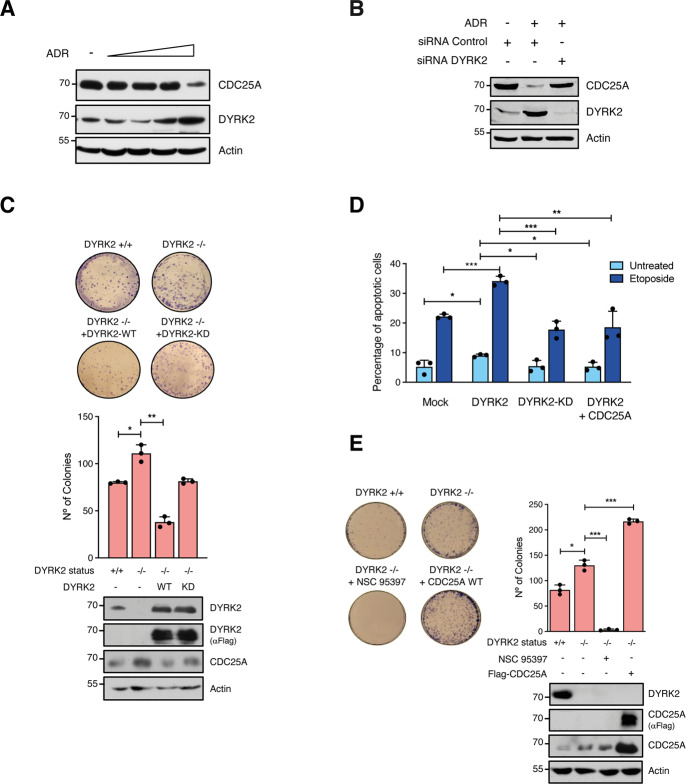
CDC25A and DYRK2 are functionally linked in response to DNA damage. **A** Lysates from MOR cells treated with increasing adriamycin (ADR) concentrations (1, 2, 4, and 6 μM) for 24 h were analyzed by WB. A representative experiment of three performed is shown. **B** HEK-293T cells were transfected with DYRK2 siRNAs or siRNA control and incubated with adriamycin (ADR; 6 μM) for 24 h after 3 days of transfection. The expression of proteins was analyzed by WB. **C** HeLa cells derived from DYRK2-KO cells and DYRK2-KO cells reconstituted with the indicated proteins were incubated with Adriamycin and viability assayed in clonogenic assays. Representative pictures are shown. Quantification of the data is shown in the bar graph (mean ± SD, *n* = 3; *P < 0.05, **P < 0.01). The expression of the indicated proteins was assessed by WB. **D** Transfected H1299 cells were treated with Etoposide (10 μM) for 24 h at 36 h post-transfection and the percentage of apoptotic cells was determined by Annexin V/PI staining (mean ± SD, *n* = 3; *P < 0.05, **P < 0.01, ***P < 0.001). **E** HeLa cells, derived DYRK2-KO cells and DYRK2-KO cells overexpressing Flag-CDC25A or pretreated with CDC25A inhibitor NSC-95397 (32 nM) for 30 min were incubated with adriamycin (3 μM) for 6 h, and viability assayed in clonogenic assays. Representative pictures are shown. Quantification of the data is shown in the bar graph (mean ± SD, *n* = 3; *P < 0.05, ***P < 0.001). The expression of the indicated proteins was assessed by WB.

It has been previously shown that CDC25A is degraded via proteasome in response to genotoxic stress and DNA damage [37, 38]. Given that DYRK2 has been placed within these pathways [39, 40], we hypothesized that they could be the context in which both proteins functionally interact. Our results showed that adriamycin (ADR) treatment increased DYRK2 expression levels in MOR and H1299 cells, concurring with published data on other cell lines [40], and with a concomitant reduction of CDC25A (Fig. 6A and Supplementary Fig. 6A). Notably, the CDC25A levels were not affected by the treatment in DYRK2 silenced cells (Fig. 6B), suggesting that the CDC25A response to the genotoxic agent is mediated by DYRK2. In addition, we observed a pro-cytotoxic effect of DYRK2 in response to ADR, since the clonogenic potential of DYRK2 knockout cells increased when compared with WT cells and was reduced upon DYRK2 reconstitution in a kinase-dependent manner (Fig. 6C). Upon genotoxic stress with a different agent as Etoposide, DYRK2 overexpression resulted in increased apoptosis in H1299 cells depending on its kinase activity (Fig. 6D), while DYRK2 depletion increased the clonogenic response to the agent (Fig. 6E). Notably, the co-expression of CDC25A inhibited the DYRK2 pro-apoptotic effect (Fig. 6D), concurring with a possible inhibitory role on DYRK2 activity and further suggesting that the effect of DYRK2 was dependent on CDC25A. To test this possibility, we used the CDC25A inhibitor NSC-95397, which showed that the increase in cell viability in DYRK2 knockout cells upon Etoposide treatment was impaired (Fig. 6E), supporting that the pro-survival activity of CDC25A and the pro-apoptotic activity of DYRK2 are functionally linked.

**Fig. 7:**
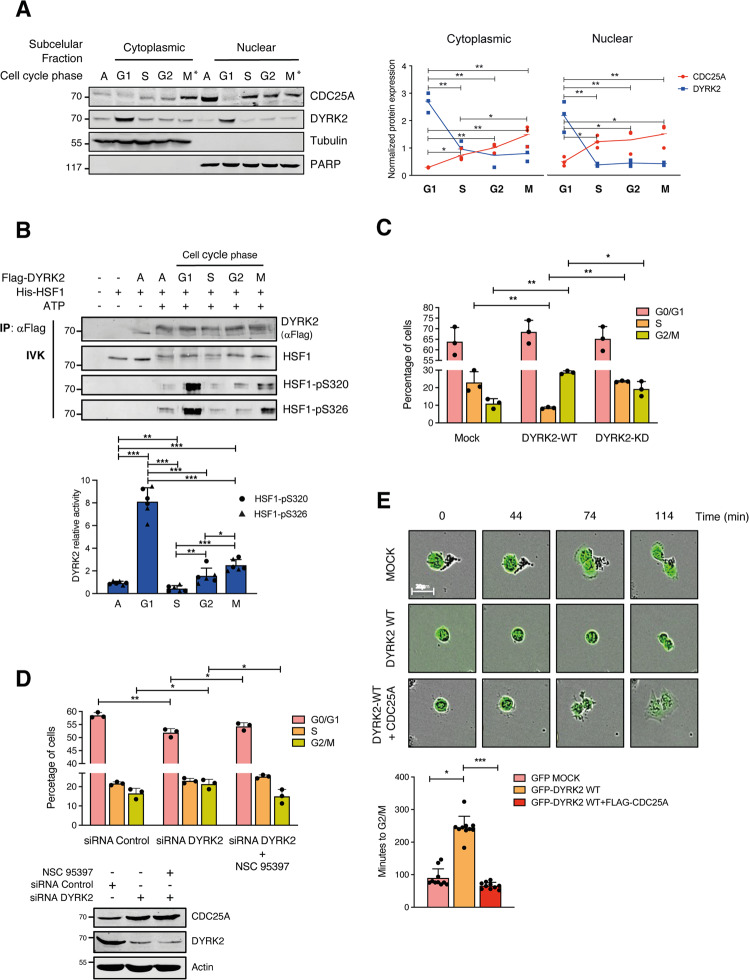
DYRK2 plays a role in cell-cycle progression through CDC25A regulation. **A** Left panel: HeLa cells were synchronized by double-thymidine block and then released into fresh media for 0 h (G1 fraction), 3.5 h (S fraction), 6 h (G2 fraction), and 10 h (M fraction) with cell cycle profiles evaluated by flow cytometry; A asynchronous cells. Nuclear and cytoplasmic fractions were analyzed by WB with Tubulin and Poly (ADP-ribose) polymerase (PARP) as markers of cytoplasmic and nuclear enrichment, respectively. A representative blot of three independent experiments performed is shown. Right panel: the graph shows the quantification of DYRK2 and CDC25A in the different phases of the cell cycle (normalized by tubulin and PARP, with the cytoplasmic level of the asynchronous cells set as 1). Right panel: the graph shows the quantification of DYRK2 and CDC25A in the different phases of the cell cycle (normalized by tubulin and PARP and with the level of the asynchronous cells set as 1) (mean ± SD, *n* = 3; *P < 0.05, **P < 0.01,) (*) M*, for cell in mitosis the cytoplasmic and nuclear fractions represent the soluble and particulate fractions, respectively. **B** HeLa cells were transfected with a Flag-DYRK2 plasmid and synchronized by double-thymidine block after 48 h. An IVK assay was performed with equal amounts of Flag-immunoprecipitated DYRK2 from the indicated fractions and recombinant HSF1 as substrate (125 ng). Protein levels of DYRK2, HSF1, and phospho-HSF1 were analyzed by WB. The upper panel is a representative blot of three independent experiments and the bottom panel shows the quantification of phospho-HSF1 at S320 and S326 after normalization with total HSF1 and DYRK2, with the level of the asynchronous cells set as 1. (mean ± SD, *n* = 3; *P < 0.05, **P < 0.01, ***P < 0.001). **C** Cell-cycle status of HeLa cells transfected with the indicated plasmids (mean ± SD, *n* = 3; *P < 0.05, **P < 0.01). **D** Cell cycle profiles of HeLa cells transfected with DYRK2 or control siRNAs and treated with the CDC25A inhibitor NSC-95397 (64 nM) for 30 min after 48 h (mean ± SD, *n* = 3; *P < 0.05, **P < 0.01). **E** H1299 cells were transfected with the indicated plasmids, and after 24 h observed by time-lapse microscopy for 24 h measuring GFP intensity. Images were captured every 4 min. Scale bar, 20 µm. Representative images from time-lapse movies at the indicated times after a cell division. Low panel: bar graph showing the quantification of the duration of the cell cycle between two cell divisions (mean ± SD, *n* = 10 independent cells; *P < 0.05).

An inverse correlation between DYRK2 and CDC25A protein amounts was observed during the cell cycle, both in synchronization experiments by serum starvation (Supplementary Fig. 6B) or by double thymidine blockade (Fig. 7A). Indeed, a detailed analysis of the subcellular localization of both proteins showed a dynamic behavior of both proteins during the cell cycle, with a clear increase in nuclear accumulation of CDC25A in S and G2 and a concomitant reduction in DYRK2 levels (Fig. 7A and Supplementary Fig. 6C). In addition, a significant increase in DYRK2 levels both in the nucleus and in the cytoplasm in the G1 phase was detected, which was accompanied by a drastic decrease in CDC25A levels (Fig. 7A). The analysis of DYRK2 kinase activity during the cell cycle also showed a maximum peak in the G1 phase (Fig. 7B), which would suggest that the increase in both DYRK2 protein levels and its enzymatic activity could contribute to the strong reduction in CDC25A accumulation observed in this phase of the cell cycle. Therefore, we wonder whether DYRK2 affected cell cycle progression. Overexpression of DYRK2 caused a significant decrease of cells in the S phase together with an increased accumulation in the G2/M phase in a kinase-dependent manner (Fig. 7C and Supplementary Fig. 6D), pointing to an impairment of the G1/S phase transition and/or exit from mitosis. The CDC25A dependence of the DYRK2 effect on the cell cycle was analyzed in H1299 cells arrested in mitosis with nocodazole. The expression of DYRK2 induced a slower mitotic exit compared to control cells, which was prevented by co-expression of CDC25A (Supplementary Fig. 6E). Next, we evaluated the effect of silencing DYRK2 in the presence of the CDC25A inhibitor NSC-95397. Cells knocked down for DYRK2 showed a clear reduction in the percentage of cells in G0/G1 together with an accumulation in the G2/M phase, which was partially recovered in the presence of NSC-95397 (Fig. 7D) Finally, the DYRK2-dependent alteration in the duration of the cell cycle was confirmed by time-lapse microscopy: cells with DYRK2-WT overexpression exhibited a slower mitotic exit, which was inhibited by the co-expression of CDC25A (Fig. 7E). These data collectively support that DYRK2 plays a role in cell-cycle progression through CDC25A regulation. These results, together with those in response to genotoxic agents, demonstrate that an inverse correlation exists between DYRK2 and CDC25A at the protein level in response to different stimuli. Moreover, they also show that this correlation is important for the DYRK2 and CDC25A-associated activities in different physiological contexts.

## Discussion

CDC25A is essential for proper cell division due to its key role in controlling the cell cycle and as a mediator of checkpoint responses to DNA damage. The best-studied post-translational mechanism for CDC25A regulation is its degradation through the ubiquitin-proteasome pathway [4], mediated mainly by SCF^βTRCP^ and APC/C-Cdh1 ubiquitin-ligase complexes [13, 30]. For its ubiquitination to happen, CDC25A is previously phosphorylated on specific residues. The upstream kinases described to date are CHK1 and CHK2, p38 MAPK, CK1α, and NEK11 [2, 38, 41, 42]. Our results indicate that DYRK2 is a novel regulator of CDC25A stability through the ubiquitin/proteasome pathway. Furthermore, the reduction of CDC25A levels observed under DNA damage, was partially restored by silencing DYRK2. These results suggest the possibility of a dual role for DYRK2 in regulating CDC25A levels, both under normal conditions and under DNA damage, as is the case for CHK1, CHK2, or p38 [2, 4].

Over the last decade, it has become evident the engagement of DYRK2 in control mechanisms for proper cell division through the phosphorylation and subsequent degradation of diverse substrates. DYRK2 regulates c-Jun and c-Myc levels during the G1 phase [21] and Tert and Katanin/60 levels during G2/M [43, 44]. Moreover, DYRK2 phosphorylates the proteasome subunit Rpt3 in a cell cycle-dependent manner, mainly during the S and G2/M phases [39]. Our results show that DYRK2 directly phosphorylates CDC25A on at least 7 residues, thereby increasing the list of DYRK2 substrates involved in the control of the cell cycle. Unexpectedly, DYRK2 promotes the degradation of CDC25A mutants in domains required for its degradation by its known ubiquitin ligases, ruling out the involvement of these domains in the process, and further suggesting a new alternative mechanism of action dependent on an E3 ligase yet to be identified. Notably, the mutation of all residues identified as phosphorylated by DYRK2 in CDC25A to a non-phosphorylatable residue led to the complete inhibition of DYRK2-mediated CDC25A degradation, which indicates that CDC25A needs to be phosphorylated by DYRK2 in more than one residue to achieve its complete degradation. Fluctuations in CDC25A protein levels during the cell cycle are the result of tight regulation. At the end of mitosis and during G1, CDC25A protein levels are kept low by APC/C-Cdh1, after which they are increased due to its requirement for the G1/S transition. During S and G2, CDC25A levels are regulated by SCF^βTCRP^-dependent turnover and finally, during mitosis, CDC25A is stabilized by Cyclin B/CDK1 allowing the G2-M transition [45]. Our results show that DYRK2 is regulated during the cell cycle, not only at the protein level but also in its enzymatic activity, opening new avenues for investigating regulatory mechanisms for this kinase. The DYRK2 dynamic behavior inversely correlates with CDC25A protein levels at any cell cycle phase, making it difficult to assess whether DYRK2 effects on CDC25A are specific to a particular cell cycle phase. Interestingly, CDK1 phosphorylates CDC25A on S18, S283 and S321 [46–48], residues that have been identified to be phosphorylated by DYRK2 in our study. Phosphorylation of CDC25A on S283 by DYRK2 could suggest a role for DYRK2 complementary to the CDK/cyclin complexes’ action during mitotic exit. An alternative, but not excluding scenario, would place DYRK2 as a key contributor to maintain low levels of CDC25A in G1. Further research is needed for a complete understanding of this mechanism.

Our results show that DYRK2 is autophosphorylated outside its T-loop. Previous studies have demonstrated that autophosphorylation regulates different aspects of DYRK family members such as protein stability, kinase activity or activity towards specific substrates [49–51]. The location of the phosphorylated residues within the 3D structure uncovers the presence of a densely phosphorylated zone close to the catalytic domain, with a possible modulatory role of the kinase activity. In fact, we observed changes in activity towards some of the DYRK2 substrates as CDC25A or NOTCH1. In addition, some of the autophosphorylated residues might regulate the subcellular localization of the kinase, which could also contribute to the activity of DYRK2 towards its substrates. Interestingly, mutation of several of these residues to non-phosphorylatable amino acids are found in cancer samples (S483G and S483R in breast ductal carcinoma; T484P in clear cell renal cell carcinoma; S499P in malignant melanoma; Cosmic v92; [52]), which might indicate that the phosphorylation status of DYRK2 is relevant for its activity in tumor cells. Finally, DYRK2 is dephosphorylated by CDC25A, suggesting a feedback regulatory loop. This represents a new control mechanism for DYRK2 and opens new pathways for its modulation through the control of the CDC25A activity.

Alterations in DYRK2 expression have been found in multiple human tumor tissues, both with higher and lower levels than in healthy tissue [18–20]. In general, low DYRK2 expression correlates with shorter survival [53], invasiveness [21], cancer recurrence [54] or poor prognosis [55]. Moreover, DYRK2 has been proposed as a potential prognostic marker for cancer and metastasis [56] and as a predictive marker for clinical responses to cancer treatment [57]. In contrast, CDC25A is considered an oncogene since it is highly overexpressed in most cancer tissues, where proliferation is increased, leading to genomic instability [2, 58]. The suggested opposed roles of the two proteins in tumor progression are consistent with the results shown here, with both proteins acting as mutual negative regulators. In agreement, the analysis of the expression levels of CDC25A and DYRK2 in different cancer types with available normal-tumor matched data identified the opposite behavior of both genes. For instance, DYRK2 expression was significantly down-regulated in colorectal tumors whereas CDC25A showed increased expression in comparison with healthy tissue; similarly, DYRK2 expression was higher in kidney tumors than in adjacent normal tissues whereas CDC25A showed the opposite pattern (Supplementary Fig. 7A, B).

**Fig. 8:**
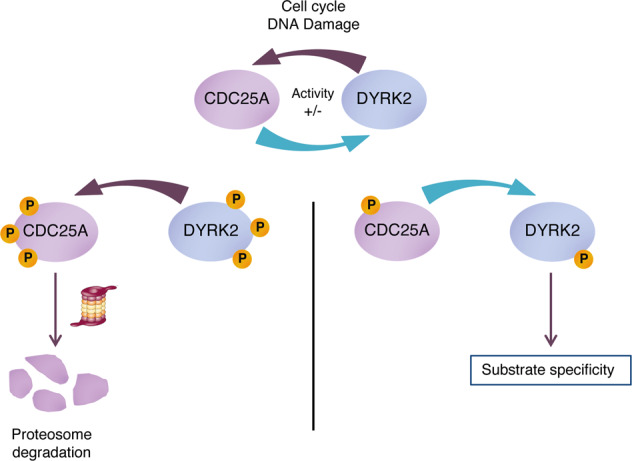
Schematic model for the crosstalk between CDC25A and DYRK2. The cell cycle or stimuli such as DNA damage are able to modulate the functional interaction between CDC25A and DYRK2. On the one hand, DYRK2 mediates CDC25A phosphorylation and its targeting to ubiquitin/proteasome-dependent degradation. On the other hand, CDC25A may dephosphorylate DYRK2 altering its specificity toward other substrates.

In summary, we describe new regulation mechanisms between two key proteins, DYRK2 and CDC25A, during normal cell cycle progression and in the context of DNA damage (Fig. 8). Whereas DYRK2 induce CDC25A degradation by a new SCF^βTRCP^ and APC/C-Cdh1-independent pathway, the phosphatase represents the first description of a regulatory mechanism for DYRK2 phosphorylation. This new switch mechanism has important implications on the control of apoptosis and cell cycle regulation.

## Material and methods

### Cell culture, transfection, plasmids, and reagents

HEK-293T (wt/DYRK2^−/−^), HeLa (wt/DYRK2^−/−^), MDA-MB-468 (wt/DYRK2^−/−^), MDA-MB-231 (wt/DYRK2^−/−^), MOR, CHO, A549, and H1299 cells were maintained in Dulbecco’s modified Eagle’s medium (DMEM). H727 and H1299 were maintained in Roswell Park Memorial Institute (RPMI) Medium. Both DMEM and RPMI were supplemented with 10% fetal calf serum, 2 mM L-glutamine, and 1% (v/v) penicillin/streptomycin (Sigma-Aldrich, St Louis, Missouri, USA) at 37 °C in a humidified atmosphere containing 5% CO_2_. See Supplementary Methods for the BEAS-2B squamous-cell differentiation model. Cell lines were routinely tested for mycoplasma and cross-contamination. Cell lines validation was performed by a multiplex PCR with Geneprint10 System (Promega, Madison, Winsconsin, USA). HEK-293T, HeLa, CHO, and H727 cells were obtained from ATCC (LGC Standards, Teddington, Middlesex, UK). MOR cells were purchased from Sigma-Aldrich. The generation of CRISPR/Cas9-cell lines was previously described [24, 25]. Transient transfections were carried out with Roti-Fect (Carl Roth, Karlsruhe, Germany), and cells were harvested between 36 and 48 h after transfection. DNA amounts in each transfection were kept constant by the addition of an empty expression vector. DYRK2 isoform 1 expression plasmids were previously described or generated by standard cloning techniques or site-directed mutagenesis. The DYRK2 kinase-dead versions (DYRK2-KD) refer to a mutation in the ATP binding site K178M. All the constructs generated were confirmed by DNA sequencing. The different reagents and plasmids used in the article are listed in Supplementary Table 1.

### Cell lysis, western blotting, and antibodies

Soluble extracts were obtained by resuspending cells in lysis buffer (50 mM Tris-HCl pH 7.5, 150 mM NaCl, 1% [v/v] NP-40, 10% [v/v] glycerol, 10 mM NaF, 1 mM Na_3_VO_4_, aprotinin [10 μg/ml], leupeptin [10 μg/ml], pepstatin [1 μg/ml] and 1 mM phenylmethylsulfonyl fluoride [PMSF]). After centrifugation, the supernatants were mixed with SDS sample buffer (50 mM Tris-HCl pH 6.8, 100 mM DTT, 2% [v/v] SDS, 0.1% [w/v] bromophenol blue and 10% [v/v] glycerol) and boiled at 95 °C. Proteins were resolved on SDS-PAGE gels and blotted to polyvinylidene difluoride membranes using a semi-dry transfer. After blocking with non-fat milk or bovine serum albumin (BSA) in Tris-buffered saline-0.1% Tween-20, primary antibodies were added. Appropriate secondary antibodies coupled to horseradish peroxidase were detected by an enhanced chemiluminescence system (USB). The different antibodies employed in the article are listed in Supplementary Table 1.

### Immunoprecipitation

Cells were washed in phosphate-buffered saline (PBS) and collected by centrifugation. The cell pellet was lysed in IP buffer (50 mM Hepes pH 7.5, 50 mM NaCl, and 1% Triton X-100) supplemented with 5 mM EGTA, 20 mM Na_4_P_2_O_7_, 50 mM NaF, 1 mM Na_3_VO_4_, 2 mM PMSF, and 10 μg/ml of leupeptin, aprotinin and pepstatin. Cell lysates were incubated with 1 μg of the indicated antibodies for 6 h at 4 °C. Antibodies were then isolated with 25 μl of protein A/G Sepharose (Santa Cruz, sc-2003), and the immunoprecipitates were washed five times in IP buffer and eluted in 1.5X SDS sample buffer. Samples were analyzed by immunoblotting.

### In vitro kinase (IVK) analysis

Commercial CDC25A and DYRK2, or bacterially expressed and purified GST-DYRK2 (WT and KD), were incubated in kinase buffer (20 mM Hepes pH 7.5, 10 mM MgCl_2_, 1 mM DTT) and 50 μM ATP plus 2.5 μCi [γ-^32^P]-ATP (3000 Ci/mmol, Amersham Biosciences) for 30 min at 30 °C. The incorporation of ^32^P was determined by SDS-PAGE and exposing the dried gel to film. For IVKs using a peptide as substrate, 200 μM DYRKtide was added to the IVK reaction, and activity was calculated as described [59]. For determination, of phosphosites by mass spectrometry (MS) (see Supplementary Methods for full details), 0.1 mM ATP was used and incubation was for 60 min at 30 °C. For some experiments, immunocomplexes of DYRK2 expressed in cells were used as the source of the kinase, and His-tagged HSF1 bacterially expressed and purified.

### Cell cycle and apoptosis assays

For cell cycle analysis, cells were fixed in 70% cold ethanol at −20 °C overnight. Cells were washed with PBS and stained with 1 mg/ml propidium iodide (PI) and treated with RNase A (50 U/ml) for 2 h at 37 °C in darkness. Details for the cell synchronization protocols are provided in Supplementary Methods. For apoptosis studies, cells were harvested in cold PBS and resuspended in binding buffer (10 mM Hepes pH 7.4, 140 mM NaCl, and 2.5 mM CaCl_2_). Cells were stained with Annexin V, Alexa Fluor 488 conjugate (Molecular Probes by Life Technologies, Carlsbad, California, USA) and PI. Cell cycle distribution and apoptosis were determined with a BD FACSCanto™ flow cytometer (BD Biosciences, San Jose, California, USA) using BD FACSDiva™ software.

### Immunofluorescence

Cells were grown on coverslips and 48 h after transfection fixed with 3.7% paraformaldehyde/PBS for 10 min. Cells were then permeabilized with 0.1% Triton X-100/PBS for 15 min, blocked with 3% BSA/PBS, and incubated overnight with primary antibodies. After being washed with PBS, cells were incubated with the secondary antibody for 45 min, and mounted on glass slides with mounting medium and 4′,6-diamidino-2-phenylindole (DAPI) (ThermoFisher,Waltham, Massachusetts, USA). Fluorescence images were captured by a confocal laser scanning microscope LSM 5 EXCITER (Carl Zeiss MicroImaging GmbH) using a 40X/1.30 oil objective (EC Plan-Neofluar) and ZEN 2008 software (Carl Zeiss MicroImaging GmbH). Images were analyzed using the ImageJ v1.45 software (http://rsbweb.nih.gov/ij/). The Costes’ approach [60] was employed for co-localization analysis between green (GFP-DYRK2 or Alexa 488) and red (Alexa 647) images to determine the Pearson’s correlation coefficient (R). Endogenous DYRK2 was detected with anti-DYRK2 obtained from Abcepta (Q92630, California, USA).

### Time-lapse microscopy

Cells were seeded in a 24-well Essen ImageLock plate (Essen BioScience, Ann Arbor, Michigan, USA) and transfected with the indicated plasmids. Then, cells were arrested at G2/M using Nocodazole (100 ng/mL) for 16 h. Finally, cells were washed and time-lapse live-cell imaging was performed with a cell imaging system IncuCyte HD (Essen BioScience). The cell cycle length was determined by measuring the time of the beginning of cell division from the first cycle to the second. Measurements were obtained from 10 cells in each independent experiment.

### Clonogenic survival assay

Transfected HeLa cells (WT and DYRK2^-/-^) were seeded in 24-well plates at 70% confluence, treated with Adriamycin (3 μM), and incubated for 24 h. Subsequently, 2000 treated cells were seeded in 6-well plates and incubated for 10 days. Cells were stained with Crystal violet solution (6% glutaraldehyde, 0.5% [w/v] Crystal violet) and the number of colonies (accumulations of more than 50 cells) was analyzed using the Image J software (http://imagej.nih.gov/ij/ [61]). The experiments were performed in duplicate.

### Data analysis

Data are expressed as mean ± SD. Differences were analyzed by unpaired Student’s *t*-tests. *P* < 0.05 was considered significant. Statistical analyses were performed using GraphPad Prism version 7.00 (GraphPad, San Diego, CA, USA). Images were analyzed and quantified using the ImageJ v1.45 software. To analyze gene expression for DYRK2 and CDC25A in The Cancer Genome Atlas (TCGA) cohorts, the pre-processed gene expression values were obtained using the FireBrowse R package (https://github.com/mariodeng/FirebrowseR) [62]. Then, the normalized gene expression values between tumor and normal tissue samples were compared using a paired two-sided *t*-test. The resulting P values were corrected to control the false discovery rate using the Benjamini and Hochberg approach. Details for the generation of the 3D DYRK2 structure model are provided in Supplementary Methods.

## Supplementary information

Supplemental Material CLEAN

Supplementary Table 1 CLEAN

Supplementary Figures CLEAN
