# Supplementary material for: A novel CDC25A/DYRK2 regulatory switch modulates cell cycle and survival
Source: Cell Death Differ. 2021 Aug 6;29(1):105–17. doi: 10.1038/s41418-021-00845-5 (PMC8738746; doi:10.1038/s41418-021-00845-5)
Supplement: Supplementary file 1 — Supplemental Material CLEAN [file 41418_2021_845_MOESM1_ESM.docx]

**Supplemental Material for:**

**A novel CDC25A/DYRK2 regulatory switch modulates cell cycle and survival**

Maribel Lara-Chica^1,2,3*^, Alejandro Correa-Sáez^1,2,3*^, Rafael Jiménez-Izquierdo^1,2,3^, Martín Garrido-Rodríguez^1,2,3^, Francisco J Ponce^1,2,3^, Rita Moreno^4^, Kimberley Morrison^4^, Chiara Di Vona^5,6^, Krisztina Arato^5,6^, Carla Jiménez-Jiménez^1,2,3^, Rosario Morrugares^1,2,3^, M Lienhard Schmitz^7^, Susana de la Luna^5,6,8,9^, Laureano de la Vega^4^ and Marco A. Calzado^1,2,3^

^1^ Instituto Maimónides de Investigación Biomédica de Córdoba (IMIBIC), Córdoba, Spain.

^2^ Departamento de Biología Celular, Fisiología e Inmunología, Universidad de Córdoba, Córdoba, Spain.

^3^ Hospital Universitario Reina Sofía, Córdoba, Spain.

^4^ Division of Cellular Medicine, School of Medicine, University of Dundee, Scotland, UK.

^5^ Centre for Genomic Regulation (CRG), The Barcelona Institute of Science and Technology (BIST), 08003, Barcelona, Spain.

^6^ Centro de Investigación Biomédica en Red en Enfermedades Raras (CIBERER), Barcelona, Spain.

^7^ Institute of Biochemistry, Justus-Liebig-University, Member of the German Center for Lung Research, D-35392, Giessen, Germany.

^8^ Universitat Pompeu Fabra (UPF), 08003, Barcelona, Spain.

^9^ Institució Catalana de Recerca i Estudis Avançats (ICREA), 08010, Barcelona, Spain.

^*^ These authors contributed equally

**Corresponding Author:** Marco A. Calzado Ph.D.

Instituto Maimónides de Investigación Biomédica de Córdoba (IMIBIC)

Avda. Menendez Pidal s/n. 14004, Córdoba, Spain

Phone: + 34 957213762

e-mail: mcalzado@uco.es

1. **Supplemental Methods**
2. **References**
3. **Supplemental Figure Legends**
   - Supplemental Figure 1: supporting data for Figure 1
   - Supplemental Figure 2: supporting data for Figure 2
   - Supplemental Figure 3: supporting data for Figure 3
   - Supplemental Figure 4: supporting data for Figure 4
   - Supplemental Figure 5: supporting data for Figure 5
   - Supplemental Figure 6: supporting data for Figure 6
   - Supplemental Figure 7: supporting data for Figure 7
4. **Supplemental Methods**

**mRNA extraction and RT-qPCR**

Cells were harvested and washed twice with phosphate-buffered saline (PBS). RNA was extracted using the High Pure RNA Isolation Kit (Roche Molecular Biochemicals, Mannheim, Germany). RNA integrity was checked on an agarose gel (Bio-Rad Universal Hood II). mRNA retrotranscription was performed with the iScript cDNA Synthesis kit (Bio-Rad, Madrid, Spain). Real-time PCR was employed with iQ™ SYBR^®^ Green Supermix (Bio-Rad) in an iCYCLER detection system (Bio-Rad). Amplification efficiencies were validated and normalized against hypoxanthine-guanine phosphoribosyltransferase (HPRT), and fold change in gene expression was calculated using the 2^−ΔΔCt^ method.

***Primer sequences:***

CDC25A-F: 5'-GCACTCGGTCAGTGTTGAAG-3'

CDC25A-R: 5'-CATGGGCCTTCTCTGGATTA-3'

HPRT-F: 5’-AATTATGGACAGGACTGAACGTCTTGCT-3’

HPRT-R: 5’-TCCAGCAGGTCAGCAAAGAATTTATAGC-3’

**BEAS-2B squamous-cell differentiation model**

BEAS-2B cells were purchased from SIGMA (European Collection of Cell Cultures, Salisbury, UK, 95102433) and maintained in serum free LHC-9 medium (Life Technologies, Carlsbad, CA, USA) at 37 °C in a humidified atmosphere containing 5% CO_2_. Squamous differentiation was acquired after culturing in LHC-9 medium enriched with 10% fetal bovine serum (FBS) for 7 days.

**Cell synchronization**

HeLa cells were synchronized in the G1 phase of the cell cycle by double thymidine block. For the first blockade, cells were incubated with 2 mM thymidine for 18 h. Then, cells were washed with 1X PBS and incubated with fresh media for 9 h. For the second blockade, cells were incubated with 2 mM thymidine for 15 h. Finally, cells were released by washing with 1X PBS and addition of fresh DMEM. Cells were harvested after 0 h (G1 fraction), 3.5 h (S fraction), 6 h (G2 fraction) and 10 h (M fraction). Evaluation of the cell cycle phases was performed by flow cytometry. For G2/M arrest, transfected H1299 cells were treated with 100 ng/μl Nocodazole (Sigma-Aldrich, St Louis, MO, USA) for 16 h, followed by mitotic release with fresh medium. To estimate the change in mitotic progression, DNA content was estimated in asynchronized and nocodazole-released cells by propidium iodide (PI) staining.

**Protein structure modelling**

The 3D DYRK2 structure model was generated using the I-TASSER server (https://zhanglab.ccmb.med.umich.edu/I-TASSER/) [1] using the DYRK2 amino acid sequence Q92630-2 from UniProt (www.uniprot.org), and the partial crystal structure of DYRK2 3K2L from the Protein Data Bank (PDB; www.rscb.org). The different predictive models were classified according to their values in 3 parameters commonly used to determine the closeness between the generated model and the structure adopted by the native protein (C-score, TM-score, and the root of the error Middle Quadratic), and we choose the best model according to these parameters. Molecular graphics and analyses were performed with University of California San Francisco (UCSF) ChimeraX [2], developed by the Resource for Biocomputing, Visualization, and Informatics at the UCSF, with support from National Institutes of Health R01-GM129325 and the Office of Cyber Infrastructure and Computational Biology, National Institute of Allergy and Infectious Diseases.

**Nucleus/Cytoplasm Subcellular Fractionation**

Cells were resuspended in 500 μl of buffer A (10 mM Hepes/KOH pH 7.9, 0.1 mM EDTA, 0.1 mM EGTA, 10 mM KCl, 1 mM β-mercaptoethanol), incubated for 5 min on ice, and further lysed by gently vortexing including 15 μl of 10% NP-40. The resuspension was centrifuged at 16,100 x*g*  for 10  s to pellet nuclei, and the supernatant representing the cytoplasmic fraction was collected. The nuclear pellet was washed in buffer A 5 times by centrifugation, and then resuspended in 100 μl of high-salt buffer B (20 mM Hepes/KOH pH 7.9, 400 mM NaCl, 1 mM EDTA, 1 mM EGTA, 1 mM β-mercaptoethanol). Finally, the solution was sonicated, centrifuged at 16,100 x*g* and the supernatant collected (nuclear fraction). All preparations were performed on ice, and protease (cOmplete™ Protease Inhibitor Cocktail, Roche) and phosphatase inhibitors (10 mM NaF and 1 mM Na_3_VO_4_) were freshly added to all buffers.

**Mass spectrometry (MS) analysis**

**Sample preparation:** Protein samples from in vitro kinase (IVK) assays were cleaned by protein precipitation with trichloroacetic acid (TCA)/acetone and solubilized in 50 μl of 0.2% RapiGest (Waters, Milford, MA, USA) in 50 mM ammonium bicarbonate. Total protein was quantified using Qubit Protein Assay Kit (Thermo Fisher Scientific, Waltham, Massachusetts, USA). Protein samples (50 μg) were incubated with 5 mM DTT at 60 °C for 30 min, and then with 10 mM iodoacetamide at room temperature for 30 min in darkness. Sequencing Grade Modified Trypsin (Promega, Madison, Wisconsin, USA) was added (ratio 1:40 trypsin:protein) and samples were incubated at 37 °C for 2 h. Trypsin was added again (ratio 1:40) and samples were incubated at 37 °C for 15 h. RapiGest was suppressed by precipitation with 0.5% trifluoroacetic acid (TFA) at 37 °C for 1 h and centrifugation. The final volume was adjusted with milliQ water and acetonitrile (ACN) to a final concentration of 0.5 μg peptide/μl (2.25% ACN and 0.2% TFA), and 1× of the iRT peptides (Biognosis AG, Schlieren/Zurich, Switzerland) were spiked in each sample.

**LC-MS analysis**: Samples were analyzed using an LTQ-Orbitrap Fusion Lumos mass spectrometer (Thermo Fisher Scientific, San Jose, California, USA) coupled to an EASY-nLC 1000 (Thermo Fisher Scientific (Proxeon), Odense, Denmark). Peptides were loaded directly onto the analytical column and were separated by reversed-phase chromatography using a 50-cm column with an inner diameter of 75 μm, packed with 2 μm C18 particles spectrometer (Thermo Scientific, San Jose, California, USA). Chromatographic gradients started at 95% buffer A and 5% buffer B with a flow rate of 300 nl/min for 5 min and gradually increased to 25% buffer B and 75% buffer A in 52 min and then to 40% buffer B and 60% buffer A in 8 min. After each analysis, the column was washed with 10% buffer A and 90% buffer B for 10 min (buffer A: 0.1% formic acid in water; buffer B: 0.1% formic acid in 80% ACN). The mass spectrometer was operated in positive ionization mode with nanospray voltage set at 2.4 kV and source temperature at 275 °C. Ultramark 1621 for the was used for external calibration of the FT mass analyzer prior the analyses, and an internal calibration was performed using the background polysiloxane ion signal at m/z 445.1200. The acquisition was performed in data-dependent acquisition (DDA) mode and full MS scans with 1 micro scan at a resolution of 120,000 were used over a mass range of m/z 350-1,500 with detection in the Orbitrap mass analyzer. Auto gain control (AGC) was set to 1E5 and charge state filtering disqualifying singly charged peptides was activated. In each cycle of data-dependent acquisition analysis, following each survey scan, the most intense ions above a threshold ion count of 10,000 were selected for fragmentation. The number of selected precursor ions for fragmentation was determined by the “Top Speed” acquisition algorithm and a dynamic exclusion of 60 seconds. Fragment ion spectra were produced via high-energy collision dissociation (HCD) at normalized collision energy of 28% and they were acquired in the ion trap mass analyzer. AGC was set to 1E4, and an isolation window of 1.6 m/z and a maximum injection time of 200 ms was used. Digested bovine serum albumin (New England Biolabs cat # P8108S) was analyzed between each sample to avoid sample carryover and to assure stability of the instrument and QCloud (REF) was used to control instrument longitudinal performance.

**Data analysis:** The spectra acquired were analyzed with the Proteome Discoverer software suite (v2.3, Thermo Fisher Scientific) and the Mascot search engine (v2.6, Matrix Science) [3]. The data was used to search the SwissProt human database (February 2020), including a list of common contaminants and the corresponding decoy entries [4]. For peptide identification, a precursor ion mass tolerance of 7 ppm was used for MS1, with trypsin as the chosen enzyme and up to three miscleavages allowed. The fragment ion mass tolerance was set to 0.5 Da for MS2. Oxidation of methionine and N-terminal protein acetylation were used as variable modifications, whereas carbamidomethylation on cysteine was set as a fixed modification. In the analysis of phosphorylated peptides, phosphorylation of serine, threonine and tyrosine were also set as variable modifications. False discovery rate (FDR) was set to a maximum of 5% in peptide identification.

**Peptide Array**

Overlapping dodecapeptides covering the entire DYRK2 and CDC25A protein sequence were spotted in an automated process on cellulose membranes. The membranes were blocked in non-fat milk in Tris-buffered saline (TBS) and incubated with GST-CDC25A or GST-DYRK2, using GST as a control, for 6 h at room temperature. After extensive washing, anti-CDC25A or anti-DYRK2 antibodies were added, respectively, and incubated for 1 h at room temperature. After extensive washing with TBS, the membranes were incubated with secondary antibodies coupled to horseradish peroxidase for 1 h at room temperature. Finally, blots were revealed by enhanced chemiluminescence using Clarity™ Western ECL Blotting Substrate (BIO-Rad).

1. **References**
2. Yang J, Zhang Y. I-TASSER server: new development for protein structure and function predictions. *Nucleic Acids Res* 2015, **43**(W1)**:** W174-181.
3. Goddard TD, Huang CC, Meng EC, Pettersen EF, Couch GS, Morris JH*, et al.* UCSF ChimeraX: Meeting modern challenges in visualization and analysis. *Protein Sci* 2018, **27**(1)**:** 14-25.
4. Perkins DN, Pappin DJ, Creasy DM, Cottrell JS. Probability-based protein identification by searching sequence databases using mass spectrometry data. *Electrophoresis* 1999, **20**(18)**:** 3551-3567.
5. Beer LA, Liu P, Ky B, Barnhart KT, Speicher DW. Efficient quantitative comparisons of plasma proteomes using label-free analysis with MaxQuant. *Methods Mol Biol* 2017, **1619:** 339-352.
6. **Supplementary Figure Legends**

**Supplementary Figure S1.** **CDC25A protein levels are modulated by DYRK2.** (**A**) Extracts from HEK-293T cells transfected with a Flag-DYRK2 expression plasmid were analyzed by WB. (**B**) HEK-293T cells were transfected to express the indicated combination of proteins. Protein expression was evaluated by immunoblot with the indicated antibodies. We show a representative blot of three independent experiments. (**C**) HEK-293T were transfected with siRNAs to DYRK2 or control siRNA and incubated with cycloheximide (CHX; 100 μg/ml) during 30, 60 and 90 min after 4 days of culture. Cell lysates were analyzed by WB with the indicated antibodies. The graph represents the mean ± SD of CDC25A band intensity from 3 different experiments. *P < 0.05, ***P < 0.001.

**Supplementary Figure S2. CDC25A stability is proteasome and DYRK2 kinase activity-dependent.** (**A**) Differentiated BEAS-2B (**B**) and H727 cells were co-transfected to express CDC25A together with DYRK2-WT or DYRK2-KD (in the presence or absence of MG-132). Cell lysates were analyzed by WB with the indicated antibodies. We show a representative WB of three independent experiments. (**C**) HEK-293T cells were transfected with plasmids to express CDC25A and DYRK2-WT or DYRK2-KD as indicated and after 36 h, treated with the protein synthesis inhibitor cycloheximide (CHX; 100 μg/ml) for 3 and 6 h. Cell lysates were analyzed by immunoblot using actin expression as the loading control. The graph represents the mean ± SD of band intensity from 3 different experiments. ***P < 0.001. (**D**) HEK-293T cells were transfected with plasmids expressing CDC25A-WT, a DSG mutant (serine 88 to alanine), a mutant with the KEN-box deleted (ΔKEN) or a version with the two mutations together, including DYRK2 or control siRNAs (100 μM). Cell extracts were analyzed by WB. We show a representative experiment of three performed.

**Supplementary Figure S3. DYRK2 co-localizes and interacts with CDC25A.** (**A**) The sequence of the peptides giving positive signal in the peptide array screen corresponding to Figure 3A and 3B is shown. (**B, C**) CHO cells were transfected with a plasmid to express GFP-DYRK2 to analyze DYRK2 co-localization with endogenous CDC25A (**B**) or together with a plasmid to express FLAG-CDC25A to analyze the co-localization of the co-expressed proteins (**C**) by indirect immunofluorescence and confocal visualization. Cells were treated with etoposide (ETP; 10 μM) or vehicle for 24 h, and with MG-132 (10 μM) for the last 4 h to promote CDC25A stabilization. Nuclear DNA was stained with DAPI. RGB profiles correspond to the fluorescence intensity through the white line indicating GFP-DYRK2 and CDC25A localization in both control and DNA damage conditions. A representative picture with overlapping localization in yellow is shown. (Pearson’s coefficient = 0.1 and Manders’ coefficients of A = 0.42; B = 0.04). Scale bar is 20 µm. Representative pictures of three independent studies are shown.

**Supplementary Figure S4. DYRK2 phosphorylates CDC25A**. Left panel, CDC25A protein sequence showing the peptides identified in the MS analysis, boxed in grey, with the phosphorylated amino acids labeled in red. Right panel, Representative spectra for the phosphorylated peptides found in CDC25A.

**Supplementary Figure S5. Autophosphorylation of DYRK2.** (**A**) DYRK2 protein sequence (corresponding to the short isoform or isoform 1, Q92630-2, NP_003574) showing the peptides identified in the MS, with the phosphorylated amino acids in red. Representative spectra for the phosphorylated peptides found in DYRK2. (**B**) HEK-293T cells were transfected with plasmids to express GFP-DYRK2 WT or the 10A mutant version (10 mutated residues), and after 36 h immunoprecipitated using an anti-GFP antibody. The immunopurified proteins were incubated in *in vitro kinase* buffer in the presence or not of ATP and analyzed by immunoblot. We show a representative blot of two independent experiments. Lanes 1 to 4 were also showed in Figure 5A. (**C**) Extracts from HeLa cells expressing Flag-DYRK2-WT or the indicated mutant versions were analyzed by WB. A representative experiment of n = 3 is shown. (**D**) CHO cells were transfected with GFP-DYRK2 or the indicated mutant versions and analyzed for the subcellular localization of the GFP fusion by direct fluorescence. Nuclear DNA was stained with DAPI. A representative image is shown for each protein. (**E**) The graph represents the ability of the DYRK2 mutants to phosphorylate the DYRKtide peptide, with the kinase activity of the WT protein arbitrarily set as 100. The DYRK2 catalytically inactive mutant (KD) was also included in the assay (mean ± SD, n = 3 independent experiments; *P < 0.05, ***P < 0.001).

**Supplementary Figure S6. DYRK2 regulate effects mediated by CDC25A. (A)** H1299 cells were treated with increasing ADR concentrations (0.5, 1, 2, 4 μM) for 24 h. Cell lysates were analyzed by WB with the indicated antibodies. We show a representative blot of three independent experiments. (**B**) H727 cells were arrested at G1 by serum-starvation for 48 h and then released by serum stimulation for 0, 4, 8, 12, or 24 h. Total cell lysates were analyzed by WB. (**C**) CHO cells were synchronized by double-thymidine block, released into fresh media for 0 h (G1 fraction), 3.5 h (S fraction), 6 h (G2 fraction) and 10 h (M fraction), and then analyzed for the subcellular endogenous localization of DYRK2 and CDC25A. A = asynchronous population. Nuclear DNA was stained with DAPI. Representative images are shown for each cell cycle phase. (**D**). Cell-cycle status of H1299 cells transiently expressing Flag-DYRK2-WT or Flag-DYRK2-KD (mean ± SD, n = 3; *P < 0.05, **P < 0.01). (**E**) H1299 cells expressing the indicated proteins were synchronized with nocodazole (100 ng/mL) and release for 3 h into cell cycle. Representative cell cycle profiles are shown, as well as the quantification of independent experiments for the post-release population (mean ± SD, n = 3; *P < 0.05, ***P < 0.001). The expression of the indicated proteins was assessed by WB.

**Supplementary Figure S7. Expression levels of CDC25A and DYRK2 in different cancer types.** (**A**) The heatmap colors represent the log2 fold change of expression for CDC25A and DYRK2 between normal and tumor samples (TCGA-gene expression analysis). The asterisk (*) indicates comparisons with an FDR corrected two-sided paired T-test P value ≤ 0.05. (**B**) TCGA expanded gene expression analysis. This figure is the expanded version of the summarized results shown in **A**. The boxplots represent the normalized expression values for normal (NT) and tumor tissues (TP), with lines indicating paired samples. Cancer cohorts where the gene expression changes are significant in opposite directions for DYRK2 and CDC25A are highlighted with red squares (FDR corrected paired T-test P value < 0.05).
