# Supplementary material for: A novel CDC25A/DYRK2 regulatory switch modulates cell cycle and survival
Source: Cell Death Differ. 2021 Aug 6;29(1):105–17. doi: 10.1038/s41418-021-00845-5 (PMC8738746; doi:10.1038/s41418-021-00845-5)
Supplement: Supplementary file 2 — Supplementary Table 1 CLEAN [file 41418_2021_845_MOESM2_ESM.docx]

**Antibodies, Reagents and Plasmids**

| **Antibody** | **Reference** | **Company** |
| --- | --- | --- |
| Flag (M2) | F3165 | Sigma-Aldrich |
| β-actin (AC-74) | A2228 |  |
| β-Tubulin III | T8660 |  |
| HA epitope (3F10) | 12158167001 | Roche Molecular Biochemicals |
| GFP | 11814460001 |  |
| Myc (9E10) | 11 667 149 001 |  |
| Ubiquitin (P4D1) | sc-8017 | Santa Cruz |
| CDK1 | sc-53219 |  |
| CDC25A (F6) | sc-7389 |  |
| DYRK2 (H80) | sc-66867 |  |
| CDC25A | MA5-13794 | Thermo Fisher Scientific |
| Phospho-S/T-Pro | 05-368 | Abcam |
| Notch1 | ab25374 |  |
| Phospho-HSF1-Ser 326 | ab115702 |  |
| Phospho-HSF1-Ser 320 | ab76183 |  |
| Phospho-CDK1 (Tyr15) | 4539 | Cell Signaling Technology |
| CDC25C (5H9) | 8688 |  |
| PARP | 9542 |  |
| HSF1 | ADI-SPA-901-D | Enzo Life Science |
| CDC25B | AF1649-SP | RyD Systems |
| DYRK2 | S473A | MRC Protein Phosphorylation and Ubiquitination Unit |
| Alexa Fluor 647 goat anti-mouse IgG1 | A21235 | Life technologies |
| Alexa Fluor 488 goat anti-rabbit IgG1 | **A32731** | Life technologies |
| DYRK2 | Q92630 | Abcepta |

| **Reagent** | **Reference** | **Company** |
| --- | --- | --- |
| MG-132 | BML-PI102-0005 | Enzo Life Science |
| 1NM-PP1 | A2228 | Santa Cruz Biotechnology |
| NSC-95397 | CAS 93718-83-3 |  |
| Etoposide | E1383 | Sigma-Aldrich |
| Cycloheximide | C7698 |  |
| Adriamycin/Doxorubicin | 44583 |  |
| Nocodazole | M1404 |  |
| Harmine | 286044 |  |
| LDN192960 | HY-13455 | MedChemExpress |
| siRNA non-targeting pool | D-001810-10-20 | Dharmacon |
| ON-TARGET plus SMARTpool against DYRK2 | L-004730-00-0010 |  |
| CDC25A recombinant protein | ab90763 | Abcam |
| DYRK2 recombinant protein | ab133138 |  |

| **Plasmid** | **Provider** | **Publication** |
| --- | --- | --- |
| HA-DYRK1A | S. de la Luna | [63] PMID: 12799418 |
| HA-DYRK3 | S. de la Luna | [63] PMID: 12799418 |
| HA-DYRK4 | S. de la Luna | [64] PMID: 21127067 |
| GFP-DYRK1B | W. Becker | PMID: 28743892 |
| Flag-CDC25A | Peter J. Stambrook | PMID: 21376736 |
| His-HSF1 | CJ. Caunt | PMID: 27354066 |
