# Supplementary material for: A novel CDC25A/DYRK2 regulatory switch modulates cell cycle and survival
Source: Cell Death Differ. 2021 Aug 6;29(1):105–17. doi: 10.1038/s41418-021-00845-5 (PMC8738746; doi:10.1038/s41418-021-00845-5)
Supplement: Supplementary file 3 — Supplementary Figures CLEAN [file 41418_2021_845_MOESM3_ESM.pdf]

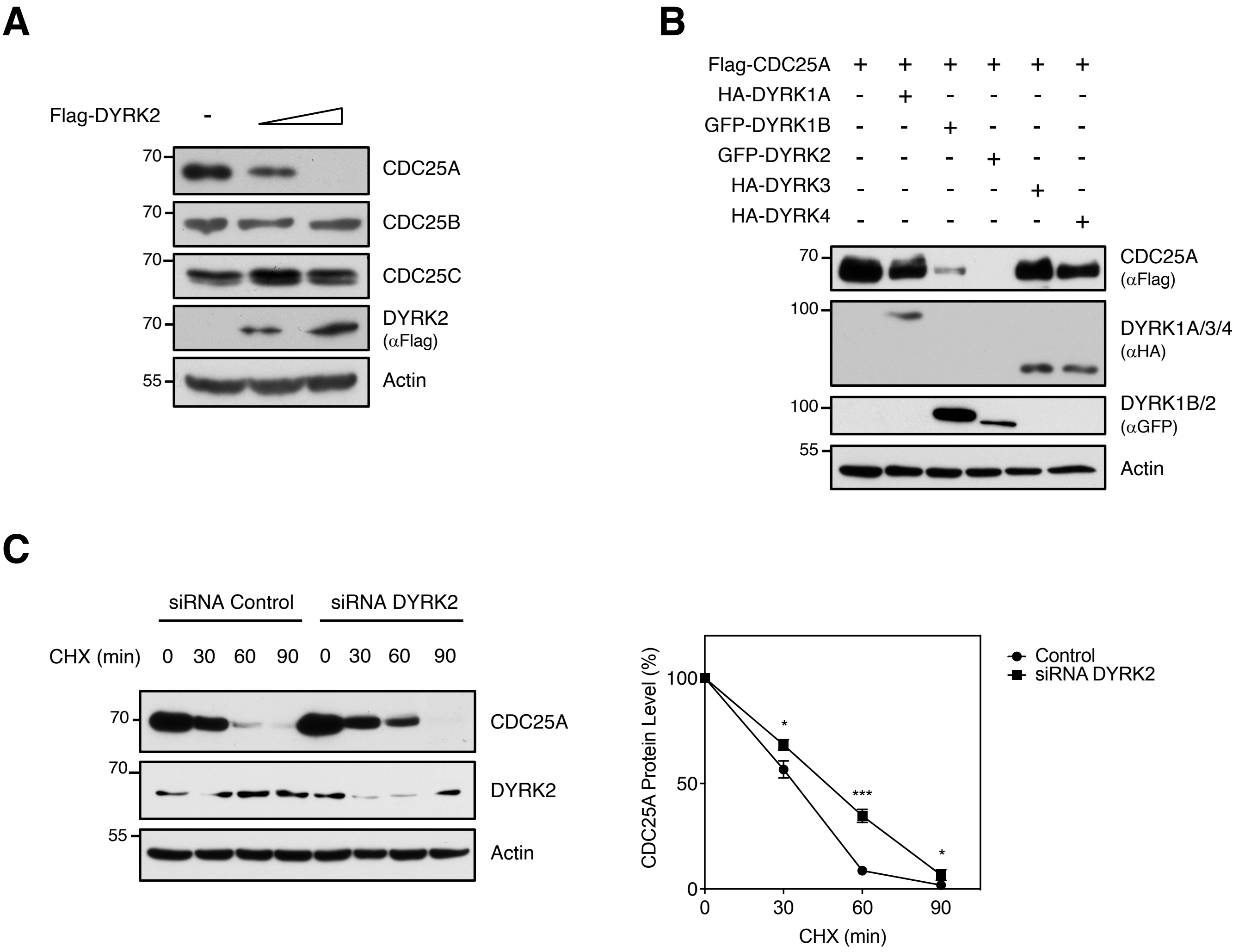
A
Flag-DYRK2
-
70
CDC25A
70
CDC25B
70
CDC25C
70
DYRK2
(αFlag)
55
Actin
B
Flag-CDC25A + + + + + +
HA-DYRK1A - + - - - -
GFP-DYRK1B - - + - - -
GFP-DYRK2 - - - + - -
HA-DYRK3 - - - - + -
HA-DYRK4 - - - - - +
70
CDC25A
(αFlag)
100
DYRK1A/3/4
(αHA)
100
DYRK1B/2
(αGFP)
55
Actin
C
siRNA Control
siRNA DYRK2
CHX (min) 0 30 60 90 0 30 60 90
70
CDC25A
70
DYRK2
55
Actin
CDC25A Protein Level (%)
100
50
0
0 30 60 90
CHX (min)
Control
siRNA DYRK2
*
***
*

SFigure 1

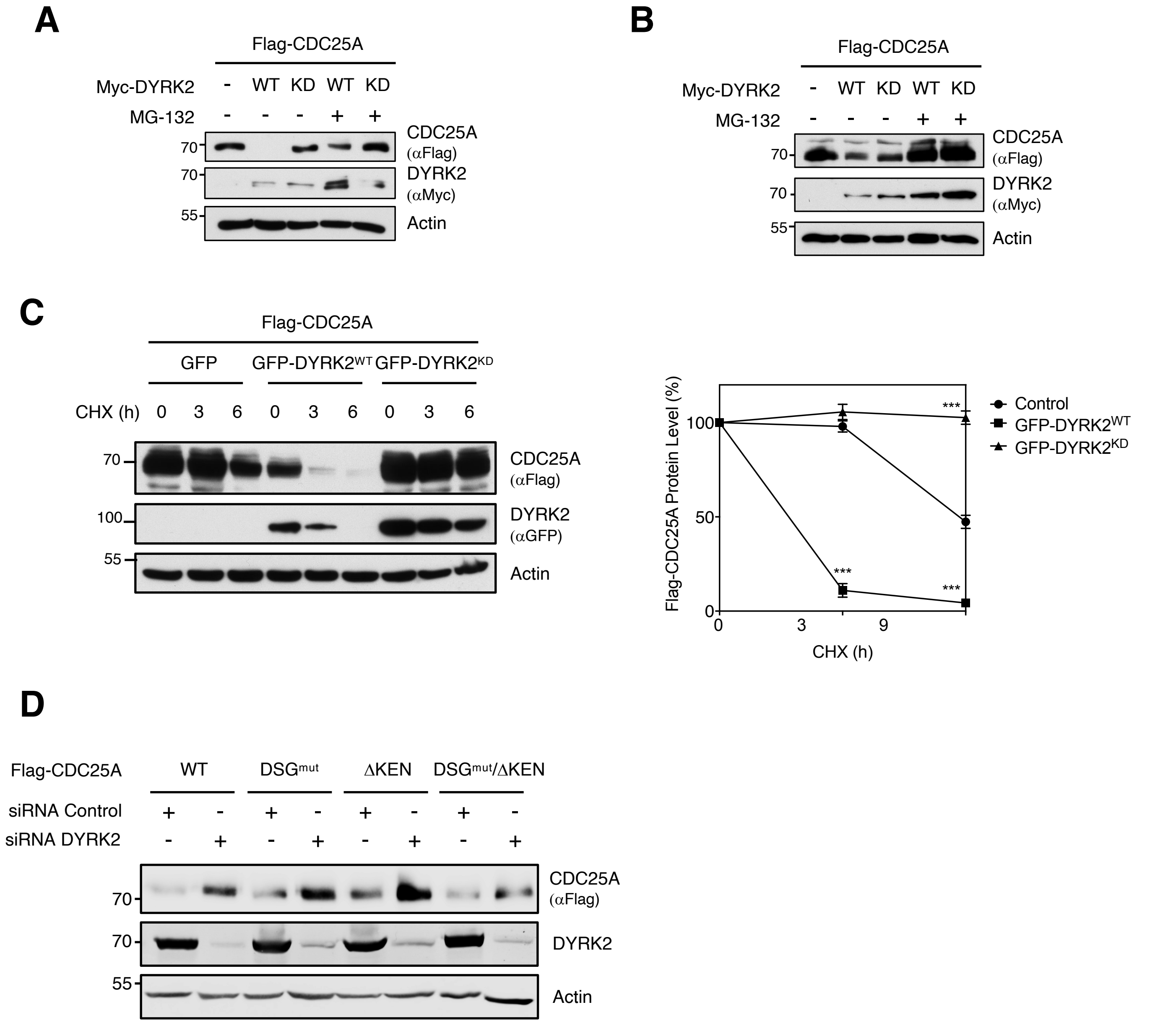
A
Flag-CDC25A
Myc-DYRK2 - WT KD WT KD
MG-132 - - - + +
70
CDC25A (αFlag)
70
DYRK2 (αMyc)
55
Actin
B
Flag-CDC25A
Myc-DYRK2 - WT KD WT KD
MG-132 - - - + +
70
CDC25A (αFlag)
70
DYRK2 (αMyc)
55
Actin
C
Flag-CDC25A
GFP GFP-DYRK2WT GFP-DYRK2KD
CHX (h) 0 3 6 0 3 6 0 3 6
70
CDC25A (αFlag)
100
DYRK2 (αGFP)
55
Actin
Flag-CDC25A Protein Level (%)
100
50
0
0 3 9
CHX (h)
***
***
***
Control
GFP-DYRK2WT
GFP-DYRK2KD
D
Flag-CDC25A WT DSGmut ΔKEN DSGmut/ΔKEN
siRNA Control + - + - + - + -
siRNA DYRK2 - + - + - + - +
70
CDC25A (αFlag)
70
DYRK2
55
Actin

SFigure 2

A

CDC25A

4 GPEPPHRRRLLF
PPHRRRLLFACS
RRRLLFACSPPP
LLFACSPPPASQ
ACSPPPASQPVV **27**

337 LIGDFSKGYLFH
DFSKGYLFHTVA
KGYLFHTVAGKH
LFHTVAGKHQDL **357**

373 FANLIKEFVIID
LIKEFVIIDCRY
EFVIIDCRYPYE
IIDCRYPYEYEG
CRYPYEYEGGHI **396**

421 DGKRVIVVFHCE
RVIVVFHCEFSS
VVFHCEFSSERG

FSSERGPRMCRY
ERGPRMCRYVRE
PRMCRYVRERDR
CRYVRERDRLGN **453**

DYRK2

4 HLHVGSHAHGQI
VGSHAHGQIQVQ
HAHGQIQVQQLF
GQIQVQQLFEDN
QVQQLFEDNSNK
QLFEDNSNKRTV **30**

29 RTVLTTQPNGLT
LTTQPNGLTTVG
QPNGLTTVGKTG
GLTTVGKTGLPV **48**

81 TPEQAMKQYMQK
QAMKQYMQKLTA
KQYMQKLTAFEH
MQKLTAFEHHEI
LTAFEHHEIFSY **105**

112 GLNAKKRQGMTG
AKKRQGMTGGPN
RQGMTGGPNNGG
MTGGPNNGGYDD
GPNNGGYDDDQG
NGGYDDDQGSYV
YDDDQGSYVQVP
DQGSYVQVPHDH
SYVQVPHDHVAY
QVPHDHVAYRYE **150**

286 QGRSGIKVIDFG
SGIKVIDFGSSC
KVIDFGSSCYEH
DFGSSCYEHQRV **306**

328 MPIDMWSLGCIL
DMWSLGCILAEL
SLGCILAELLTG
CILAELLTGYPL **348**

B

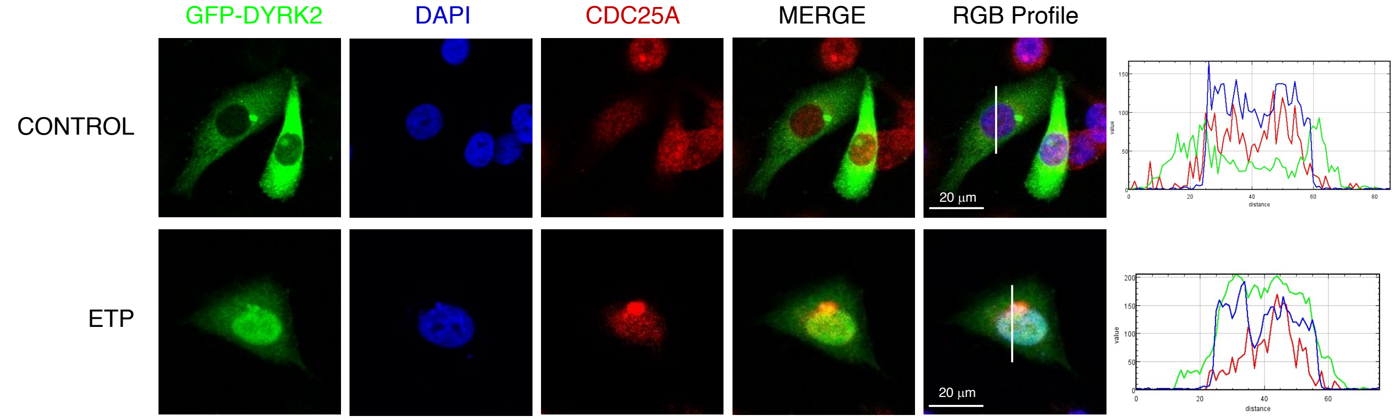

C

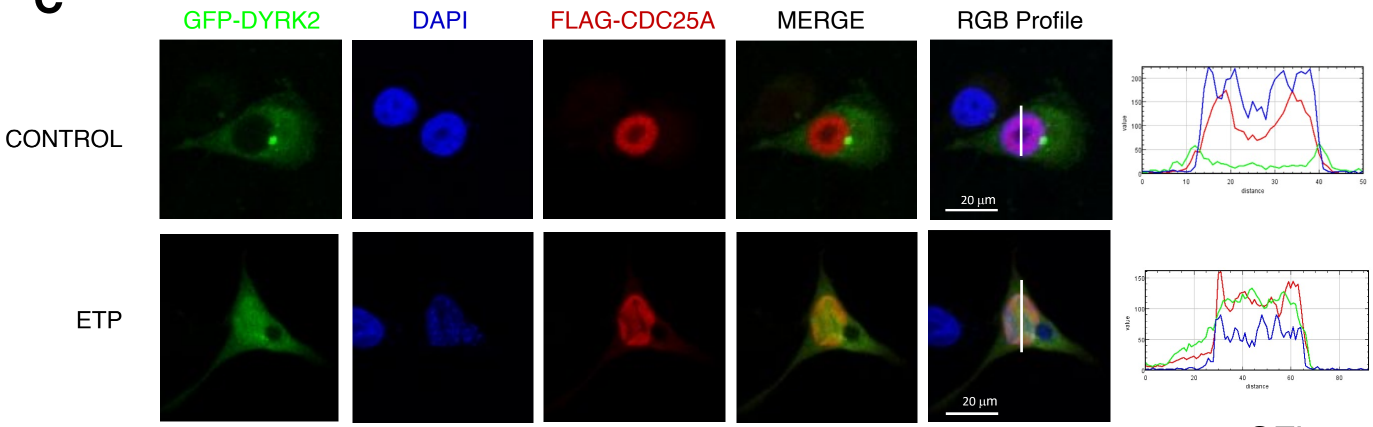

SFigure 3

```
        10         20         30         40         50
MELGPEPPHR RRLLFACSPP PASQPVVKAL FGASAAGGLS PVTNLTVTMD
        60         70         80         90        100
QLQGLGSDYE QPLEVKNNSN LQRMGSSEST DSGFCLDSPG PLDSKENLEN
       110        120        130        140        150
PMRRIHSLPQ KLLGCSPALK RSHSDSLDHD IFQLIDPDEN KENEAFEFKK
       160        170        180        190        200
PVRPVSRGCL HSHGLQEGKD LFTQRQNSAP ARMLSSNERD SSEPGNFIPL
       210        220        230        240        250
FTPQSPVTAT LSDEDDGFVD LLDGENLKNE EETPSCMASL WTAPLVMRTT
       260        270        280        290        300
NLDNRCKLFD SPSLCSSSTR SVLKRPERSQ EESPPGSTKR RKSMSGASPK
       310        320        330        340        350
ESTNPEKAHE TLHQSLSLAS SPKGTIENIL DNDPRDLIGD FSKGYLFHTV
       360        370        380        390        400
AGKHQDLKYI SPEIMASVLN GKFANLIKEF VIIDCRYPYE YEGGHIKGAV
       410        420        430        440        450
NLHMEEEVED FLLKKPIVPT DGKRVIVVFH CEFSSERGPR MCRYVRERDR
       460        470        480        490        500
LGNEYPKLHY PELYVLKGGY KEFFMKCQSY CEPPSYRPMH HEDFKEDLKK
       510        520
FRTKSRTWAG EKSKREMYSR LKKL
```

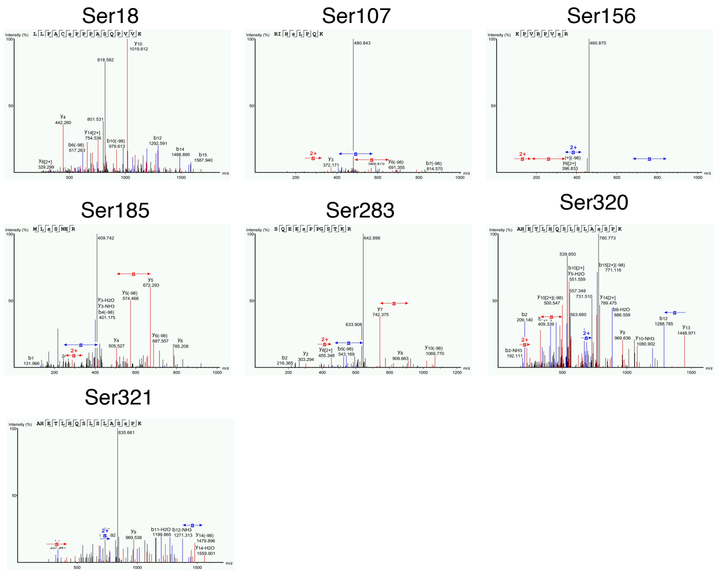

SFigure 4

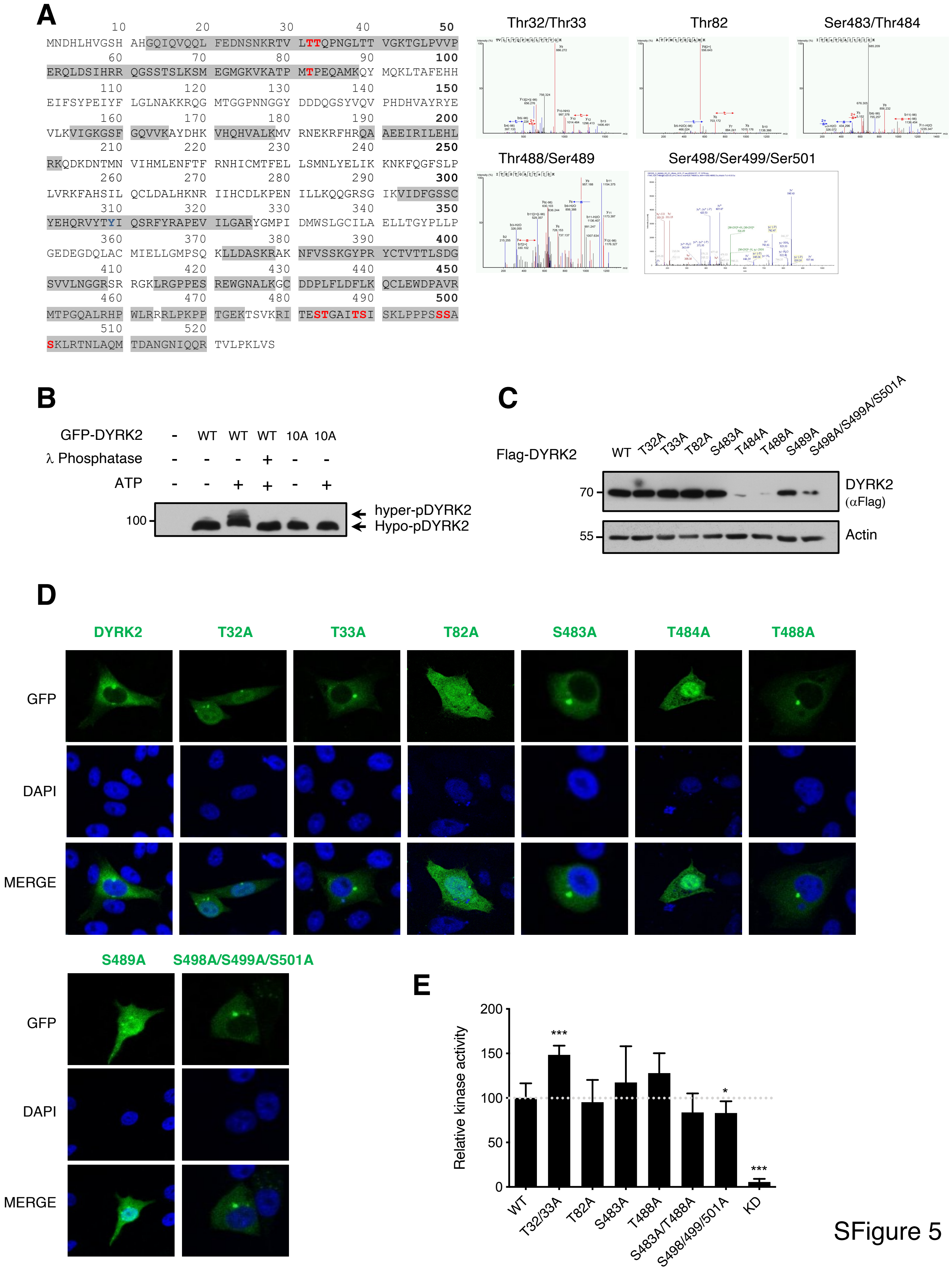
A
10 20 30 40 50
MNDHLHVGSH AHGQIQVQQL FEDNSNKRTV LTTQPNGLTT VGKTGLPVVP
60 70 80 90 100
ERQLDSIHRR QGSSTSLKSM EGMGKVKATP MTPEQAMKQY MQKLTAFEHH
110 120 130 140 150
EIFSYPEIYF LGLNAKKRQG MTGGPNNGGY DDDQGSYVQV PHDHVAYRYE
160 170 180 190 200
VLKVIGKGSF GQVVKAYDHK VHQHVALKMV RNEKRFHRQA AEEIRILEHL
210 220 230 240 250
RKQDKDNTMN VIHMLENFTF RNHICMTFEL LSMNLYELIK KNKFQGFSLP
260 270 280 290 300
LVRKFAHSIL QCLDALHKNR IIHCDLKPEN ILLKQQGRSG IKVIDFGSSC
310 320 330 340 350
YEHQRVYTYI QSRFYRAPEV ILGARYGMPI DMWSLGCILA ELLTGYPLLP
360 370 380 390 400
GEDEGDQLAC MIELLGMPSQ KLLDASKRAK NFVSSKGYPR YCTVTTLSDG
410 420 430 440 450
SVVLNGGRSR RGKLRGPPES REWGNALKGC DDPLFLDFLK QCLEWDPAVR
460 470 480 490 500
MTPGQALRHP WLRRRLPKPP TGEKTSVKRI TESTGAITSI SKLPPPSSSA
510 520
SKLRTNLAQM TDANGNIQQR TVLPKLVS
Thr32/Thr33
Thr82
Ser483/Thr484
Thr488/Ser489
Ser498/Ser499/Ser501
B
GFP-DYRK2 - WT WT WT 10A 10A
λ Phosphatase - - - + - -
ATP - - + + - +
100
hyper-pDYRK2
Hypo-pDYRK2
C
Flag-DYRK2
WT T32A T33A T82A S483A T484A T488A S489A S498A/S499A/S501A
70
55
DYRK2 (αFlag)
Actin
D
DYRK2 T32A T33A T82A S483A T484A T488A
GFP
DAPI
MERGE
S489A S498A/S499A/S501A
GFP
DAPI
MERGE
E
Relative kinase activity
200
150
100
50
0
***
*
***
WT T32/33A T82A S483A T488A S483A/T488A S498/499/501A KD

SFigure 5

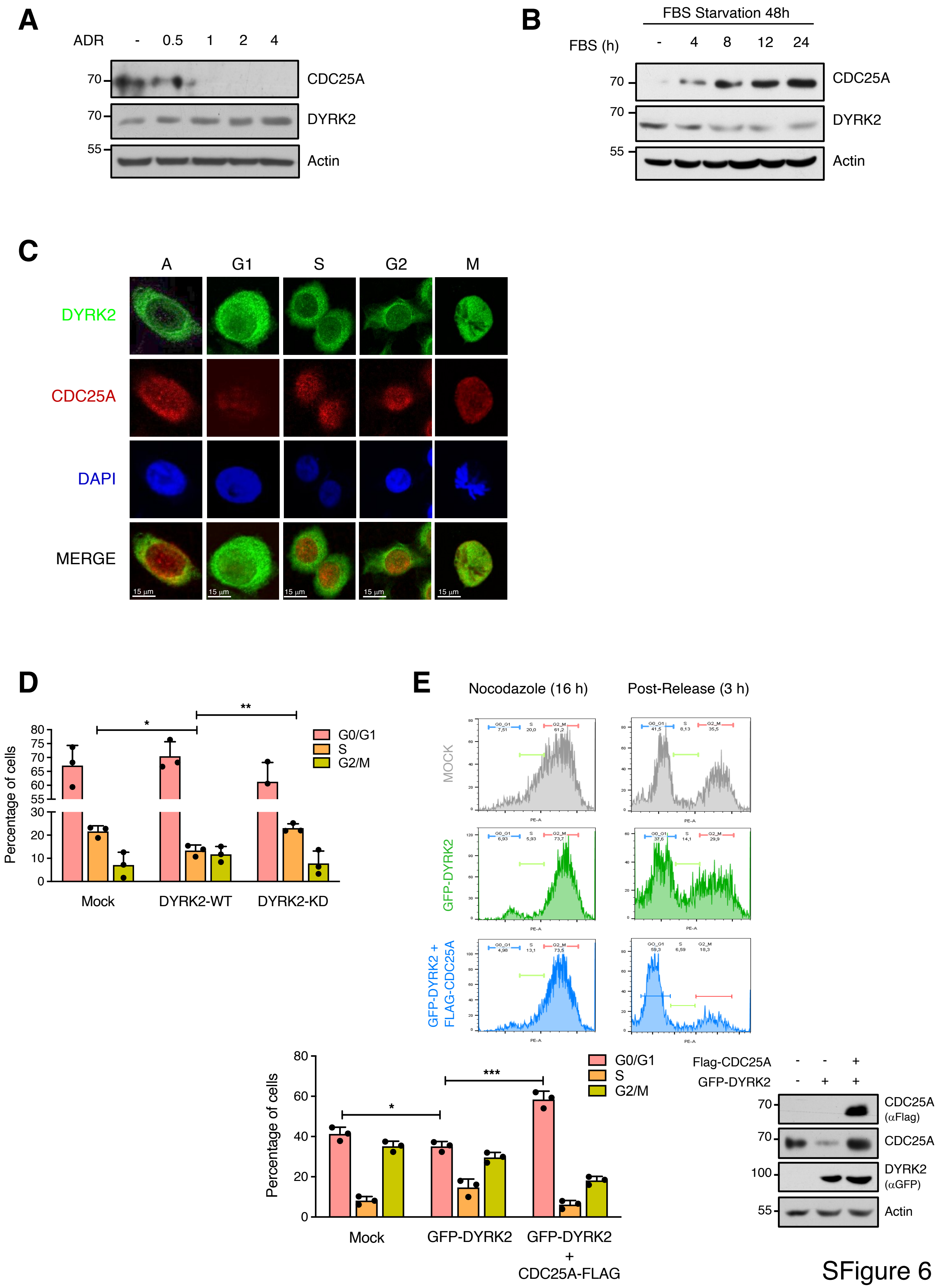
A
ADR - 0.5 1 2 4
CDC25A
DYRK2
Actin
B
FBS Starvation 48h
FBS (h) - 4 8 12 24
CDC25A
DYRK2
Actin
C
A G1 S G2 M
DYRK2
CDC25A
DAPI
MERGE
15 µm
D
Percentage of cells
G0/G1
S
G2/M
Mock
DYRK2-WT
DYRK2-KD
E
Nocodazole (16 h)
Post-Release (3 h)
MOCK
GFP-DYRK2
GFP-DYRK2 +
FLAG-CDC25A
Percentage of cells
Mock
GFP-DYRK2
GFP-DYRK2
+
CDC25A-FLAG
Flag-CDC25A - - +
GFP-DYRK2 - + +
CDC25A
(αFlag)
CDC25A
DYRK2
(αGFP)
Actin

SFigure 6

A

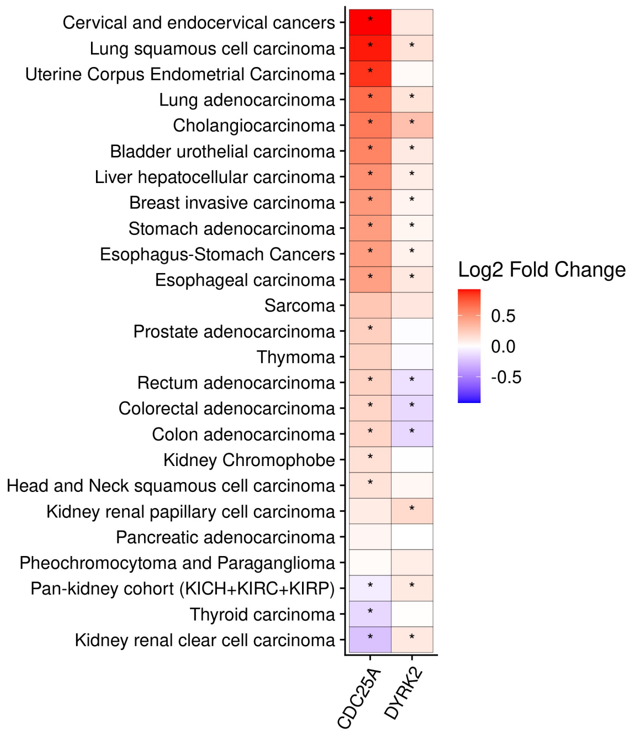
Cervical and endocervical cancers
Lung squamous cell carcinoma
Uterine Corpus Endometrial Carcinoma
Lung adenocarcinoma
Cholangiocarcinoma
Bladder urothelial carcinoma
Liver hepatocellular carcinoma
Breast invasive carcinoma
Stomach adenocarcinoma
Esophagus-Stomach Cancers
Esophageal carcinoma
Sarcoma
Prostate adenocarcinoma
Thymoma
Rectum adenocarcinoma
Colorectal adenocarcinoma
Colon adenocarcinoma
Kidney Chromophobe
Head and Neck squamous cell carcinoma
Kidney renal papillary cell carcinoma
Pancreatic adenocarcinoma
Pheochromocytoma and Paraganglioma
Pan-kidney cohort (KICH+KIRC+KIRP)
Thyroid carcinoma
Kidney renal clear cell carcinoma
CDC25A
DYRK2
Log2 Fold Change
0.5
0.0
-0.5

B

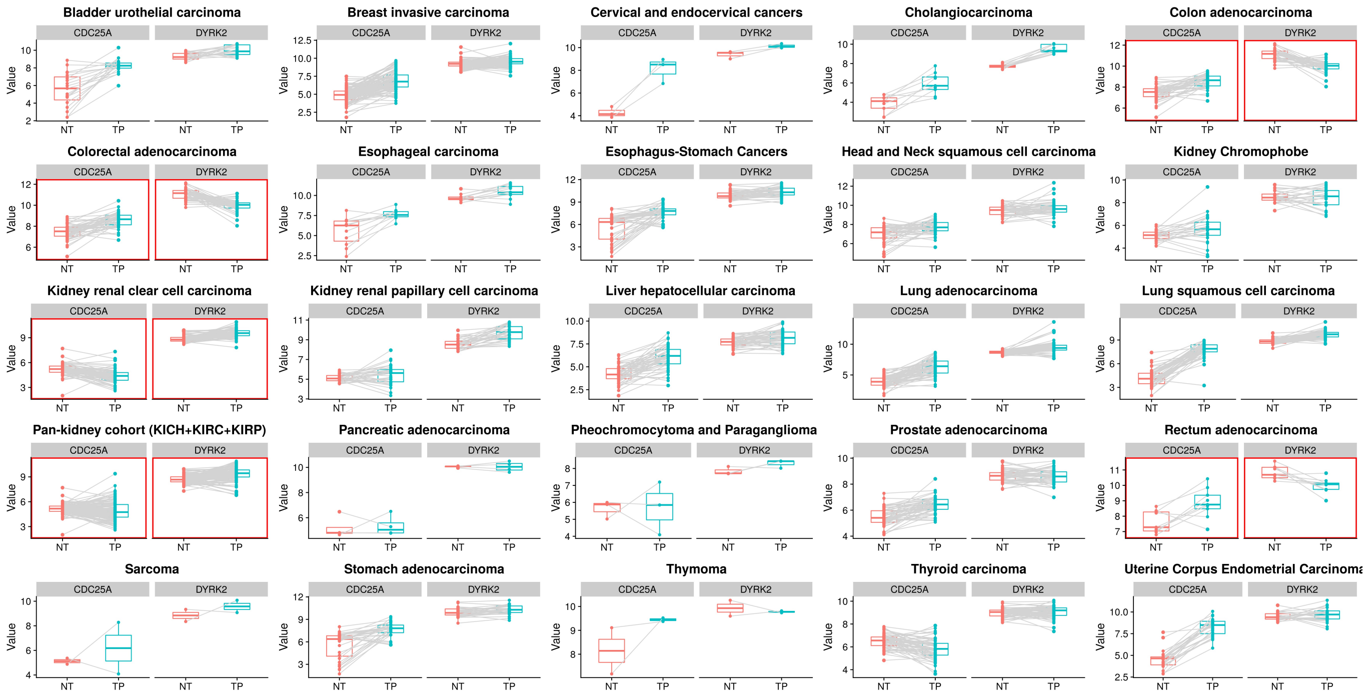
Bladder urothelial carcinoma
Breast invasive carcinoma
Cervical and endocervical cancers
Cholangiocarcinoma
Colon adenocarcinoma
Colorectal adenocarcinoma
Esophageal carcinoma
Esophagus-Stomach Cancers
Head and Neck squamous cell carcinoma
Kidney Chromophobe
Kidney renal clear cell carcinoma
Kidney renal papillary cell carcinoma
Liver hepatocellular carcinoma
Lung adenocarcinoma
Lung squamous cell carcinoma
Pan-kidney cohort (KICH+KIRC+KIRP)
Pancreatic adenocarcinoma
Pheochromocytoma and Paraganglioma
Prostate adenocarcinoma
Rectum adenocarcinoma
Sarcoma
Stomach adenocarcinoma
Thymoma
Thyroid carcinoma
Uterine Corpus Endometrial Carcinoma
CDC25A
DYRK2
Value
NT
TP

SFigure 7
